# The *Rhodococcus equi* virulence protein VapA disrupts endolysosome function and stimulates lysosome biogenesis

**DOI:** 10.1002/mbo3.416

**Published:** 2016-10-19

**Authors:** Adam P. Rofe, Luther J. Davis, Jean L. Whittingham, Elizabeth C. Latimer‐Bowman, Anthony J. Wilkinson, Paul R. Pryor

**Affiliations:** ^1^ Department of BiologyWentworth WayUniversity of YorkYorkUK; ^2^ Cambridge Institute for Medical Research and Department of Clinical BiochemistryAddenbrooke's HospitalUniversity of CambridgeCambridge; ^3^ Structural Biology LaboratoryDepartment of ChemistryUniversity of YorkYorkUK; ^4^ Hull York Medical SchoolUniversity of YorkYorkUK

**Keywords:** endolysosome, lysosome, *Rhodococcus equi*, VapA

## Abstract

*Rhodococcus equi* (*R. equi*) is an important pulmonary pathogen in foals that often leads to the death of the horse. The bacterium harbors a virulence plasmid that encodes numerous virulence‐associated proteins (Vaps) including VapA that is essential for intracellular survival inside macrophages. However, little is known about the precise function of VapA. Here, we demonstrate that VapA causes perturbation to late endocytic organelles with swollen endolysosome organelles having reduced Cathepsin B activity and an accumulation of LBPA, LC3 and Rab7. The data are indicative of a loss of endolysosomal function, which leads cells to upregulate lysosome biogenesis to compensate for the loss of functional endolysosomes. Although there is a high degree of homology of the core region of VapA to other Vap proteins, only the highly conserved core region of VapA, and not VapD of VapG, gives the observed effects on endolysosomes. This is the first demonstration of how VapA works and implies that VapA aids *R. equi* survival by reducing the impact of lysosomes on phagocytosed bacteria.

## 1 Introduction

*Rhodococcus equi*,* (R. equi*) is a gram‐positive facultative intracellular pathogen with similarities to *Mycobacterium tuberculosis* (Vazquez‐Boland et al., 2013). Pathogenic *R. equi* infects a wide variety of animal hosts including pigs, sheep, and cattle, but is most frequently associated with bronchopneumonial disease in foals and is increasingly isolated from immunocompromised humans (Hayes, Diaz‐Guzman, & Hoopes, 2011; Khan, Ali, & Baqi, 2013; Nath, Mathew, Mohan, & Anila, 2013; Topino, Galati, Grilli, & Petrosillo, 2010). Transmission of infection in foals is thought to occur through inhalation of contaminated dust or soil particles (Muscatello, Anderson, Gilkerson, & Browning, 2006). *R. equi* represents a major threat to foal health worldwide and has a significant economic impact on the horse breeding industry. Current treatments for *R. equi* infections involve combination drug therapies with rifampin and macrolides such as clarithromycin (Giguère et al., 2011). These treatments can be protracted and expensive, and are not always successful. Furthermore, antibiotic‐resistant strains are emerging (Burton et al., 2013). While promising potential vaccines are in development, there are still no commercially available vaccines (Cauchard et al., 2013) and research efforts are now focussing on the mechanisms of pathogenicity induced by *R. equi* in order to provide insights which may lead to better treatments for infection.

*R. equi* is phagocytosed by lung alveolar macrophages and becomes enclosed in an internal vacuole called the *R.equi‐containing vacuole* (RCV) in which the bacterium survives. The precise character of this vacuolar compartment is unclear since there are conflicting reports on whether *R. equi* reaches the degradative environment of the lysosome (Fernandez‐Mora, Polidori, Luhrmann, Schaible, & Haas, 2005; Toyooka, Takai, & Kirikae, 2005). It is therefore uncertain how *R.equi* is able to survive intracellularly. Eventually, *R. equi* begins to multiply within the RCV, resulting in necrosis of the host cell (Luhrmann et al., 2004).

The ability of *R. equi* to persist and replicate intracellularly is dependent upon the presence of ~ 90 kb virulence plasmid, which plays a crucial role in intracellular survival. Loss of the virulence plasmid renders the bacteria unable to replicate inside macrophages in vitro (Giguere et al., 1999; Hondalus & Mosser, 1994). Virulent strains are also cleared significantly faster in a mouse lung infection model than strains harboring this extra chromosomal element (Gonzalez‐Iglesias et al., 2014). A striking feature of the virulence plasmid is the presence of a pathogenicity island containing several genes encoding virulence‐associated proteins (Vaps). The expression of one of these genes in particular, *vapA*, has been associated with virulence in vivo in both mice and foals (Giguere et al., 1999; Takai et al., 1991). VapA has also been shown to be necessary, but not sufficient for virulence, since VapA alone can restore virulence to a Vap‐A, ‐C, ‐D, ‐E, and ‐F deletion mutant (Jain, Bloom, & Hondalus, 2003) but cannot restore virulence to plasmid‐cured *R. equi* strains (Giguere et al., 1999). More recently, it has been shown that *vapA* along with two other genes *virR* and *virS,* are the minimum genes required to confer virulence, in the absence of the virulence plasmid (Coulson et al., 2015). VirR is a LysR‐type transcriptional regulator (Russell, Byrne, O'Connell, Boland, & Meijer, 2004) and VirS is an OmpR/PhoB response regulator (Kakuda et al., 2014), both of which are required for proper *vapA* gene expression (Ren & Prescott, 2004). The presence of VirR and VirS alters transcription of ~18% of *R  equi* genes. This suggests that the plasmid is also needed to regulate chromosomal genes that may be required for intracellular growth (Coulson et al., 2015). VapA has also been demonstrated to be necessary to reduce fusion of *R. equi*‐containing phagosomes with lysosomes (Fernandez‐Mora et al., 2005; von Bargen et al., 2009).

Sequence alignment of the different Vap proteins shows that they share a high degree of homology in the C‐terminus with little homology in the N‐terminus of the proteins. The crystal structures of VapB, VapD, and VapG have been determined by X‐ray crystallography (Geerds, Wohlmann, Haas, & Niemann, 2014; Okoko, Blagova, Whittingham, Dover, & Wilkinson, 2015; Whittingham et al., 2014) revealing that the highly conserved C‐terminal region forms a tightly packed eight‐stranded β‐barrel and that the structures of these Vap proteins are superimposable. However, the crystal structures of the Vap proteins do not show any obvious ligand‐binding sites or grooves that would provide clues to their function. It is predicted, by homology, that VapA will have a similar structure to other Vap proteins; yet, there is nothing obvious in the VapA sequence that indicates why VapA is unique among the Vap proteins in being necessary for virulence.

Given the importance of VapA for *R. equi* intracellular survival, we sought to determine how VapA alone affects the endocytic pathway. Our data indicate that only the β‐barrel structure of VapA and not the β‐barrel structure of other Vap proteins disrupt endolysosome function, promoting cells to upregulate lysosome biogenesis to compensate for the loss of functional endolysosomes. While VapA has been shown to be important for virulence, these are the first data that demonstrate that VapA could aid *R. equi* intracellular survival by reducing cellular lysosomal function.

## 2 Experimental Procedures

### 2.1 Reagents

Mouse anti‐rat LGP120 (GM10) and anti‐rat LGP110 were generous gifts from Prof. Paul Luzio (University of Cambridge). Rabbit anti‐VapA was a kind gift from Prof. A. Haas (University of Bonn). Mouse anti‐myc antibody (9E10), rat anti‐mouse LAMP1 (1D4B), and mouse anti‐human LAMP1 (H4A3) were from the Developmental Studies Hybridoma Bank (DSHB, University of Iowa). ciMPR antibodies (ab124767) were from Abcam. Rabbit monoclonal antibodies to Rab5 (C8B1) and Rab7 (D95F2) were from Cell Signaling Technology. Anti‐LC3 (clone 5F10) was from Nanotools, anti‐LBPA (clone 6C4) was from Merck Millipore. The plasmid for producing recombinant VapAD was a kind gift from Wim Meijer (University College Dublin). All chemical reagents and the mouse monoclonal anti‐α‐tubulin were from Sigma.

### 2.2 Mammalian cell culture

All cells were cultured in Dulbecco's modified Eagle's medium (DMEM) supplemented with 10% (v/v) FBS, 2 mmol/L glutamine, and 1% (v/v) penicillin‐streptomycin (10 U/ml and 10 μg/ml, respectively) in a humidified 5% CO2 atmosphere at 37°C, unless otherwise stated. Flp‐In HeLa cells were a kind gift from Prof. M. Lowe (University of Manchester) and an isogenic cell line expressing myc‐VapA was made by cloning myc‐VapA into pCDNA5/FRT/TO and transfecting cells with this vector along with pOG44. Transfected Flp‐In HeLa cells were selected with 50 μg/ml Hygromycin B (Roche) and protein expression was induced with 1 μg/ml doxycycline for 24 hr.

### 2.3 Recombinant protein production

*E.coli* (B21 DE3 pLysS) were transformed with plasmids encoding either GST‐ or His_6_‐tagged proteins as appropriate. Bacteria were grown in 2 × 600 ml of LB or 2TY broth as follows: 7.5 ml of LB (plus the appropriate selective antibiotics) was inoculated with a single bacterial colony and grown overnight at 37°C with shaking. The next day, starter cultures were diluted 1:500 into a total volume of 600 ml LB or 2TY broth, and grown at 37°C until an OD_600_ of 0.6–2.0 was obtained. Protein production was induced with IPTG to a final concentration of 0.2 mmol/L, and cultures grown for a further 4 hr. The bacteria were pelleted at 2,645* g* for 15 min at 4°C. The cell pellet was resuspended in 10 ml of bacterial lysis buffer (1% (v/v) Triton X‐100, 1 mg/ml lysozyme, 2 mmol/L MgCl_2_, 1 U/ml DNAse, in PBS) with protease inhibitors (Roche), and frozen at −20°C overnight. The cell pellet was defrosted on ice and centrifuged at 47,800*g* in a Sorvall SS34 rotor for 20 min at 4°C. For GST‐fusion proteins, the supernatant was incubated with 1 ml (packed volume) of glutathione sepharose (GE healthcare) for 1 hr at 4°C with gentle rotation. The sepharose beads were then collected and washed with 3 × 20 ml of wash buffer (1% (v/v) Triton X‐100 in PBS). GST/GST‐fusion proteins were eluted with 20 ml elution buffer (50 mmol/L Tris, pH 8.0, 10 mmol/L reduced glutathione). These fractions were then analyzed by SDS‐PAGE. The eluted proteins were then pooled and dialysed extensively into PBS at 4°C for 2 days and stored at −20°C until needed. For the purification of His_6_‐tagged proteins, cell pellets were processed as above. The soluble supernatant was incubated with 134 μl (packed volume) of His‐Select resin (Sigma) for 1–2 hr at 4°C with gentle rotation. The beads were washed with 3 × 20 ml wash buffer (10 mmol/L imidazole, 0.3 mol/L NaCl, 50 mmol/L Na_2_HPO_4_, pH8.0). Proteins were eluted with 20 ml elution buffer (250 mmol/L imidazole, 0.3 mol/L NaCl, 50 mmol/L Na_2_HPO_4_, pH 8.0) and 1 ml fractions collected. Fractions were analyzed by SDS‐PAGE. The eluted proteins were then pooled and dialysed extensively into PBS at 4°C for 2 days and stored at −20°C until needed.

### 2.4 Cell‐feeding experiments

J774.2, Normal Rat Kidney (NRK) or HeLa cells were seeded onto glass coverslips in 24‐well plates and cultured for 24 hr. Cells were then incubated with the appropriate protein at 100 μg/ml for varying amounts of time. Cells were routinely rinsed three times with PBS and then fixed and processed for confocal microscopy as described. Where cells were fed VapA in the presence of Bafilomycin‐A1, the Bafilomycin‐A1 concentration was 100 nmol/L.

### 2.5 LysoTracker and Magic Red Cathepsin B substrate experiments

For LysoTracker experiments, NRK cells stably expressing LGP120‐GFP (a kind gift from J. P. Luzio, University of Cambridge) were seeded onto glass‐bottomed 35‐mm dishes (MatTek Corporation) before being incubated with 100 μg/ml of recombinant VapA‐His_6_ or VapD‐His_6_ protein for 24 hr. The cells were then incubated with LysoTracker (as per the manufacturer's instructions) for 5 min before the LysoTracker and LGP120 were visualized on a Zeiss 880 confocal microscope. For Magic Red Cathepsin B substrate (ABD Serotec Ltd) experiments, cells seeded onto glass‐bottomed dishes were incubated with or without VapA for 24 hr before the medium was exchanged for medium containing the Magic Red Cathepsin B substrate (as per the manufacturer's instructions). Ten minutes after the addition of the substrate, the cresyl violet fluorphore was visualized on a Zeiss 880 confocal microscope using the 561 nm laser line at 2% power. For fluorescence recovery after photobleaching (FRAP) experiments, 10 images were taken before regions of interest were bleached using 50 iterations with combined laser lines 488 nm at 4% power, 514 nm at 2% power, 561 nm at 100% power, and 633 nm at 0.2% power and then up to 300 images were subsequently taken with the 561 nm laser line at 2% power.

### 2.6 *R. equi* infection

NRK and J774.2 cells were seeded onto glass coverslips and cultured for 48 hr in antibiotic‐free DMEM medium supplemented with 10% (v/v) FBS (heat inactivated to 56°C for 30 min) and 2 mmol/L glutamine. *R. equi* was cultured overnight in BHI media with shaking at 30°C. Bacteria were surface‐labeled with Alexa Fluor‐488 as described ((Fernandez‐Mora et al., 2005). An appropriate number of bacteria were taken to infect mammalian cells at an MOI (multiplicity of infection) of 10 and resuspended in PBS. Bacteria were centrifuged onto cells at *80g* for 5 min and incubated for 1 hr at 37°C to allow phagocytosis of bacteria. Monolayers were rinsed three times with DMEM to remove unbound bacteria, and the media replaced with DMEM containing 150 μg/ml gentamycin to kill extracellular bacteria. The cells were cultured for a further hour, and washed with PBS. The media was then replaced with DMEM containing 10 μg/ml gentamycin, and cells cultured for 24 hr to allow intracellular bacteria to grow. Cells were fixed with 4% formaldehyde in PBS for 20 min at room temperature and then processed for immunofluorescence.

### 2.7 Western blotting

Proteins were transferred to nitrocellulose membranes using the iBlot system (Invitrogen) as per the manufacturer's instructions. Membranes were then blocked in either 5% (w/v) semi‐skimmed milk or 5% (w/v) BSA in TBS (100 mmol/L NaCl, 10 mmol/L Tris‐HCl, pH 7.4) + 0.1% (v/v) Tween‐20 (TBST) for 30 min at room temperature or overnight at 4°C. Membranes were probed with primary antibodies in 5% (w/v) milk or 5% (w/v) BSA TBST for 1 hr at room temperature or 4°C overnight. Membranes were then washed with TBST (3 × 5 min washes) and incubated with horseradish peroxidase‐conjugated secondary antibodies in milk or BSA at room temperature for 30 min. Membranes were washed a further three times with TBST and then proteins were visualized using ECL reagent (GE Healthcare).

### 2.8 Immunofluorescence

Cells were rinsed once with Dulbecco's PBS (Sigma) and then fixed with 4% formaldehyde in PBS for 20 min at 20°C before quenching in 50 mmol/L NH_4_Cl in PBS for 10 min. For the LC3 antibody, cells were further fixed for 10 min with −20°C MeOH at −20°C. After fixation, cells were permeabilized in 0.2% (w/v) BSA, 0.05% (w/v) Saponin in PBS, for 10 min. All further washes and antibody dilutions were carried out in BSA/Saponin/PBS (BSP) solution. In all cases, coverslips were incubated with primary antibodies diluted in BSP for 1 hr at room temperature. Coverslips were washed 3 × 5 min with BSP and then incubated with Alexa fluorophore‐conjugated secondary antibodies (diluted 1:300 in BSP) for 30 min, followed by a further 3 × 5 min washes with BSP. For visualization of DNA, 4′,6‐diamidino‐2‐phenylindole (DAPI) was included at 1 μg/ml in the final wash. Coverslips were rinsed once in distilled water, blotted dry, and mounted onto slides with MOVIOL 4–88 containing 2.5% (w/v) DABCO (1,4‐diazobicyclo [2,2,2]‐octane). Slides were viewed on a Zeiss LSM 880 inverted confocal laser microscope running Zen software (2015) or a Zeiss LSM 510 meta Axioplan 2M upright confocal laser microscope running Zen software (2009, Carl Zeiss Ltd, Germany). Gain and laser powers were set accordingly. All images are maximum intensity z‐projections unless otherwise stated. The size of puncta were determined using ImageJ and setting threshold levels to match the fluorescence, setting the scale for the image being analyzed and then using the default analyze particles option.

### 2.9 Electron microscopy

BSA‐gold (10 nm) was made as previously described (Bright, Reaves, Mullock, & Luzio, 1997). Cells were pulsed for 4 hr with BSA‐gold, washed with PBS and the gold chased for 4 hr and then cells were fed VapA for 24 hr. Cells were then fixed with 0.1mol/L Na cacodylate, pH 7.2, 2% (w/v) PFA and 2.5% (w/v) glutaraldehyde for 1 hr at 20°C. Cells were scraped and pelleted at 16,500 *g* for 10 min. Cells were post‐fixed with 1% (w/v) OsO_4_ in 0.1 mol/L sodium cacodylate buffer, pH 7.2, “en bloc” stained with 0.5% (w/v) uranyl acetate in 50 mmol/L sodium maleate buffer, pH 5.2, dehydrated in ethanol and processed for TEM in Araldite CY212 epoxy resin (Agar Scientific, Stansted, United Kingdom). Ultrathin sections (50 nm) were mounted on formvar/carbon‐coated EM grids and stained with uranyl acetate and lead citrate. Sections were examined with an FEI Tecnai G2 Spirit BioTwin transmission electron microscope (Eindhoven, The Netherlands) at an operating voltage of 80 kV and images were recorded with an Eagle 4K CCD camera.

### 2.10 Cellular fractionation

NRK cells were scraped into STM buffer (250 mmol/L sucrose, 10 mmol/L TES, pH 7.2, 1 mmol/L MgCl_2_) and then subjected to nitrogen cavitation (Parr Instrument Company) at 500psi for 10 min. STM buffer of 1 ml was used for every confluent T75 flask. The lysates were centrifuged at 1,500 *g* for 10 min and 600 μl of the postnuclear superntatant overlayed onto Nycodenz gradients consisting of 2 ml 10% (w/v) Nycodenz, 2 ml 16% (w/v) Nycodenz, and 0.5 ml 45% (w/v) Nycodenz. Nycodenz solutions were made by diluting 60% (w/v) Nycodenz with STM buffer. Gradients were centrifuged in a Beckman VTi90 rotor at 90,000 rpm for 1 hr. Gradients were pumped off from the bottom using a long needle and a peristaltic pump, collecting approximately 27 × 190 μl fractions. ß‐hexosaminidase was assayed as previously described (Pryor, 2015).

### 2.11 TFEB experiments

A TFEB‐GFP construct in a pEGFP vector was a gift from Andrea Ballabio (Telethon Institute of Genetics and Medicine, Pozzuoli, NA, Italy) and was subcloned into a pLXIN retroviral expression vector (Clontech, Mountain View, CA, USA). A clonal NRK cell line stably expressing TFEB‐GFP was generated using the pLXIN retroviral system as previously described (Gordon et al. 2009). Transduced cells of mixed expression levels were selected by culture in medium supplemented with 0.5 mg/ml geneticin (G418) and clonal NRK cell lines expressing TFEB‐GFP were subsequently generated after isolating single cells by fluorescence‐activated cell sorting. Nuclear translocation of TFEB‐GFP was quantified after fixing with 4% (w/v) paraformaldehyde and staining nuclei with Hoechst 33342 using a Cellomics ArrayScan^™^ VTi high content screening, wide‐field microscope with Cellomics ArrayScan^™^ software. After autofocussing using the Hoechst 33342 channel to detect the nuclei, the program maps and outlines the area of each nucleus (Nuc) and a region extending outward by an adjustable number of pixels (Cyt, representing a significant portion of the cytoplasm). Nuclear translocation is calculated as the NucCyt difference (AU, arbitrary units), which is mean fluorescence intensity in Nuc after subtracting the mean intensity of pixels in Cyt. (Mean ± SEM of 750 cells). Treatment with Torin 1 (250 nmol/L for 90 min) was used as a positive control for TFEB‐GFP translocation to the nucleus (Settembre et al., 2012). Images showing TFEB‐GFP localization were obtained from paraformaldehyde‐fixed cells using a Zeiss LSM 880 confocal microscope.

## 3 Results

### 3.1 VapA causes swelling of late endocytic compartments

**Figure 1:**
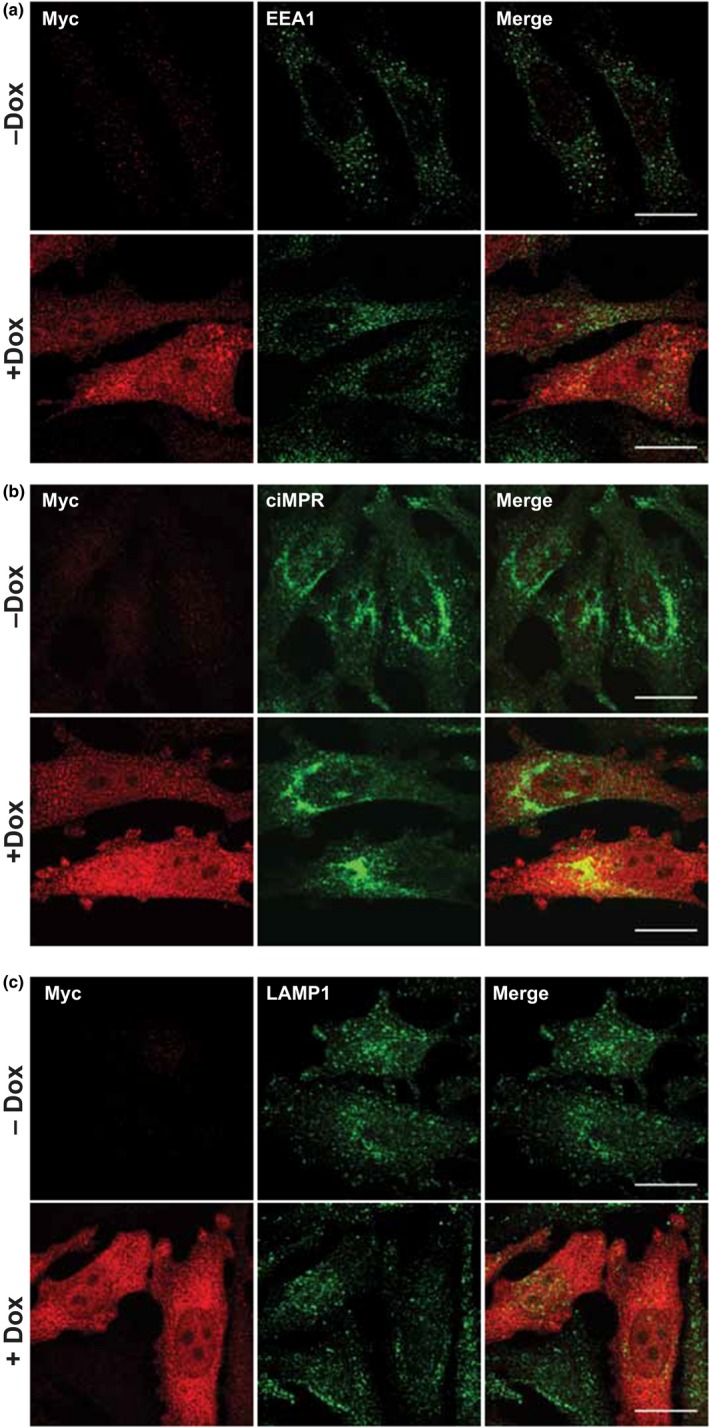
VapA expression in the cytoplasm of cells has no effect on endocytic compartments. 1 μg/ml doxycycline was added to HeLa cells to induce expression of myc‐VapA for 24 hr. Cells were fixed and double‐labeled with myc and EEA1 (**a**), ciMPR (**b**) and LAMP1 (**c**) antibodies followed by fluorescently labeled secondary antibodies. Myc labeling is shown in red and organelle labeling is shown in green. Scale bars, 20 μm

**Figure 2:**
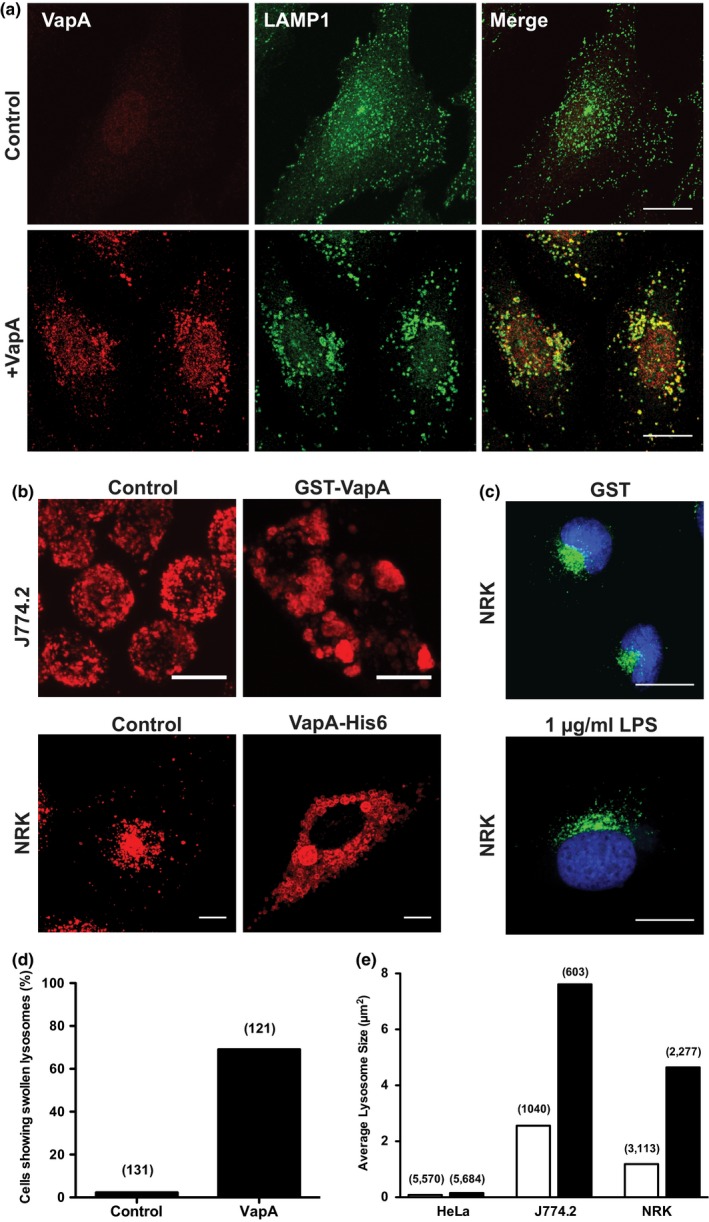
VapA induces lysosomal swelling from within the lumen of lysosomes. (a) HeLa cells were incubated with or without 100 μg/ml recombinant VapA‐His_6_ protein in the cell medium for 24 hr. Cells were then fixed and double immunolabeled with α‐VapA antibodies and α‐LAMP1 antibodies followed by fluorescently labeled secondary antibodies. VapA labeling is shown in red and LAMP1 labeling is shown in green. (b) J774.2 or Normal Rat Kidney (NRK) cells were incubated with or without 100 μg/ml recombinant GST‐VapA or recombinant VapA‐His_6_ proteins for 24 hr, respectively. Cells were then fixed and labeled with α‐LAMP1 (J774.2) or α‐LGP120 (NRK) antibodies followed by fluorescently labeled secondary antibodies. Scale bar, 10 μm (c) NRK cells were incubated with 100 μg/ml recombinant GST protein or 1 μg/ml LPS for 24 hr. Cells were then fixed and labeled with α‐LGP120 antibodies followed by fluorescently labeled secondary antibodies (green). Cell nuclei were stained with DAPI (blue). Scale bar, 20 μm. All images are confocal maximum intensity z‐projections. Wider fields of view are shown by figure S2. (d) Quantification of the number of HeLa cells showing swollen lysosomes data are from a typical experiment. Numbers in parentheses represent the number of cells that were scored. (e) Quantification of lysosomal size in HeLa, J774.2 and NRK cells in control cells (open bars) and cells fed with VapA (closed bars). Data are from a typical experiment. Numbers in parentheses represent the number of lysosomes that were counted (approximately, 60 cells per condition)

**Figure 3:**
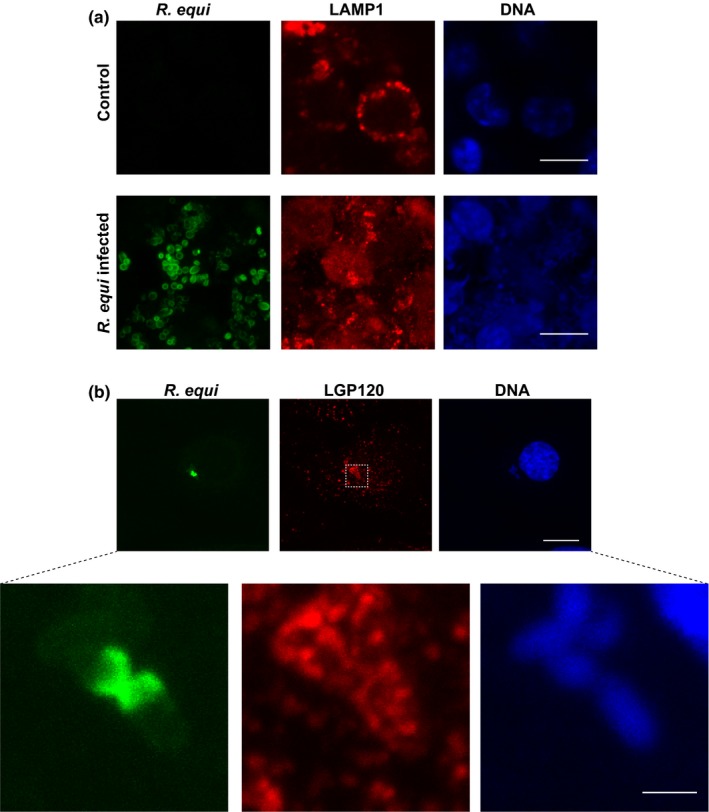
*R. equi‐*infected Normal Rat Kidney (NRK) cells show swollen lysosomal compartments. (a) J774.2 were seeded onto glass coverslips and cultured for 48 hr in antibiotic‐free Dulbecco's modified Eagle's medium (DMEM) medium. Cells were then infected with Alexa Fluor‐488 surface‐labeled *R. equi* (green) with an MOI of 10 and cells cultured for 24 hr to allow intracellular bacteria to grow. Cells were fixed and immunolabeled for LAMP1 followed by a fluorescently labeled secondary antibody (red). Cell nuclei and bacterial DNA were stained with DAPI (blue). Scale bars, 10 μm. Images are confocal maximum intensity z‐ projections. (b) NRK cells were infected as for J774.2 cells but lysosomes were immunolabeled with an antibody against LGP120. A region is highlighted which is magnified (bottom panels). Scale bar, 10 μm. Scale bar for the magnified image is 2 μm. Images were acquired using a Zeiss LSM 880 with Airyscan processing and shown with a maximum intensity z‐projection

VapA is the only Vap protein that is essential for virulence (Jain et al., 2003), yet its molecular function remains unknown. Normally, phagocytosis of bacteria results in the delivery of the bacteria to lysosomes where they are destroyed by lysosomal acid hydrolases and indeed VapA has been shown to reduce fusion of the *R. equi*‐containing phagosome with lysosomes (Fernandez‐Mora et al., 2005). However, we wanted to test the hypothesis that VapA may disrupt lysosomal function per se, thereby allowing *R. equi* to survive intracellularly. We first expressed myc‐tagged VapA (under the control of a doxycycline‐inducible promoter) for 24 hr in the cytoplasm of HeLa cells and the morphology of endocytic compartments was determined by confocal microscopy (Figure 1). EEA1, ciMPR, and LAMP1 staining (markers of early endosomes, late endosomes, and lysosomes, respectively) in cells expressing myc‐VapA were indistinguishable from that seen in control cells. Given that *R. equi* has no known secretory system for translocating effector proteins into the host cell cytoplasm (Letek et al., 2010), we also considered the possibility that VapA may aid *R. equi* survival from within the RCV. To test this hypothesis, VapA was produced recombinantly with either an N‐terminal GST tag or a C‐terminal His_6_ tag. HeLa cells, J774.2 mouse macrophages, and NRK cells were incubated with 100 μg/ml tagged VapA, to allow VapA to be taken up by fluid‐phase endocytosis. Strikingly, the late endosomes (Fig. S1) and lysosomes in these cells became enlarged and swollen (Figure 2a,b), whereas early endocytic compartments (EEA1‐positive puncta) were not affected (Fig. S1). This swollen lysosomal phenotype was not caused by LPS endotoxin (that might be present in the VapA protein preparation) or the GST tag, as GST and LPS endotoxin had no effect on lysosome morphology (Figure 2c). Therefore, the swollen lysosomal phenotype was due to the presence of VapA. Typically, over 65% of all HeLa cells treated with VapA showed swollen lysosomes (Figure 2d) with a 1.8‐fold, a threefold, and a fourfold increase in lysosomal size in HeLa, J774.2, and NRK cells, respectively (Figure 2e). Thus, we aimed to characterize the effect on late endosomes and lysosomes further. *R. equi* readily infects J774.2 cells (Figure 3a, Fig. S3) with a loss of discrete lysosomes (compared to uninfected cells), as has previously been reported (Hietala & Ardans, 1987; von Bargen et al., 2009; Zink, Yager, Prescott, & Fernando, 1987). Although macrophages are the normal host cell for *R. equi*, the available cell line (J774.2) is not ideal for confocal analysis, since all endocytic compartments are in close proximity, which hinders their analyses. Due to the spatial distribution of lysosomes in NRK cells and the greatest differences in the size of lysosomes when fed VapA, we used NRK cells for subsequent studies. While NRK cells do not normally phagocytose bacteria, they can be infected with *R. equi* (Figure 3b, Fig. S3). NRK cells are infected by *R. equi* with low efficiency compared to J774.2 cells (Fig. S3). However, the bacteria are able to replicate inside NRK cells as shown by Figure 3b, as the Alexa Fluor‐488‐labeled bacteria replicate the surface fluorescence is lost but DAPI staining of nucleic acid of replicated bacteria can be seen. At 24 hr, NRK cells show less lysis when infected with *R. equi*, compared to mouse macrophages, with no discernable loss of lysosomes, but it is unclear as to whether this is a difference in the cell type or that NRK cells are not as permissive for *R. equi* infection. However, infected NRK cells show swollen lysosomes, as visualized by LGP120 (rat equivalent of human LAMP1) immunofluorescence, (Figure 3b) mirroring the phenotype seen when VapA protein is fed to NRK, J774.2, and HeLa cells.

### 3.2 The core of VapA is sufficient to cause late endosome/lysosome swelling

**Figure 4:**
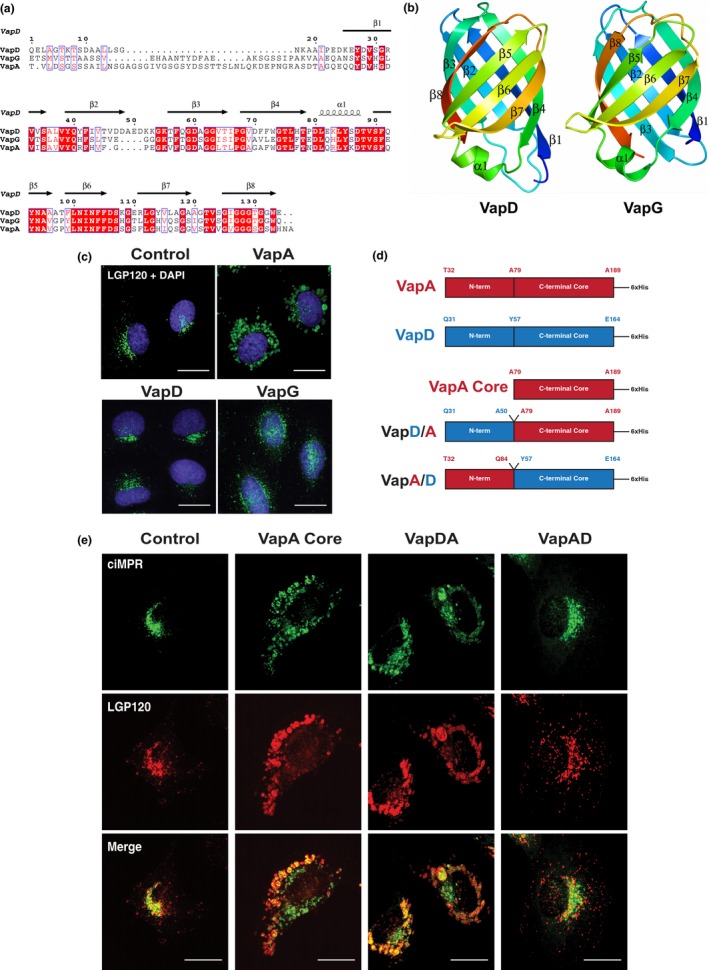
The core of VapA is sufficient to cause lysosomal swelling. (a) Alignment of the sequences of VapA, VapD, and VapG displayed together with the secondary structure elements of VapD using the program ESPRIPT (Robert & Gouet, 2014). Invariant residues in the alignment are shown in white type on a red background; conserved residues are in blue boxes. (b) Ribbon representation of the structures of VapD and VapG with the secondary structure elements labeled. In each case, the ribbon is color‐ramped from the N‐terminus (blue) to the C‐terminus (red). (c) Normal Rat Kidney (NRK) cells were incubated with 100 μg/ml of recombinant VapA, VapD, or VapG for 24 hr. Cells were then fixed and immunolabeled with LGP120 antibodies followed by Alexa Fluor‐488‐ labeled anti‐mouse secondary antibodies (green). Cell nuclei were stained with DAPI (blue). Images are representative of three separate experiments. (d) Schematic representation of recombinant chimeric Vap proteins used in cell‐feeding experiments as shown by panel C. Residue numbers correspond to the full protein sequence including the signal peptide. N‐term refers to the unstructured N‐terminal region and C‐terminal core represents the β‐barrel “core” structure. (e) NRK cells were incubated with 100 μg/ml of recombinant VapA core, VapDA, or VapAD for 24 hr. Cells were then fixed and double‐immunolabeled with LGP120 and ciMPR antibodies followed by fluorescently labeled secondary antibodies. Images are representative of two separate experiments. Scale bars, 10 μm. All images are confocal maximum intensity z‐projections. Wider fields of views of immunofluorescence images are shown by figure S4

While the amino acid sequences of the N‐terminal segment of the Vap proteins do not share a high degree of homology and are not predicted to have any tertiary structure, the remaining part of the Vap proteins (C‐terminal core structure) are remarkably conserved (Figure 4a). Given the high degree of homology between the C‐terminal core region of VapA and other Vap proteins (Whittingham et al., 2014) and their identical tertiary structures (Figure 4b), we asked whether VapD or VapG were able to induce a similar phenotype to that seen with VapA. When NRK cells were incubated with VapD‐His_6_ or VapG‐His_6_, no swelling of lysosomal compartments was observed compared to VapA (Figure 4c). Since the major difference between the Vap proteins is in the N‐terminal segment, we hypothesized that the N‐terminal region of VapA would be responsible for the observed lysosomal swelling. We therefore sought to determine whether the N‐terminus of VapA was necessary to induce the swollen lysosomal phenotype, or whether the N‐terminus of other Vap proteins was inhibitory to any potential function of their C‐terminal core region. NRK cells were incubated with VapA lacking the N‐terminal segment (VapA core) or chimeric VapA/VapD proteins (Figure 4d). After feeding cells recombinant proteins for 24 hr, the morphology of late endosomes and lysosomes was examined. Cells incubated either the VapA core protein or a VapD (N‐terminal segment)‐VapA core chimeric protein had enlarged late endosomes and lysosomes. Conversely, the late endosomes and lysosomes of cells incubated with VapA (N‐terminal region)‐VapD core protein were identical to control cells. Thus, the N‐terminal segment of VapA is not necessary for its activity and the core of VapA alone is sufficient to induce the swelling of endocytic compartments. This result was unexpected given the high degree of homology of the core region of VapA to the other Vap proteins.

### 3.3 VapA‐induced swollen compartments accumulate intralumenal vesicles, LBPA, Rab7, and LC3

**Figure 5:**
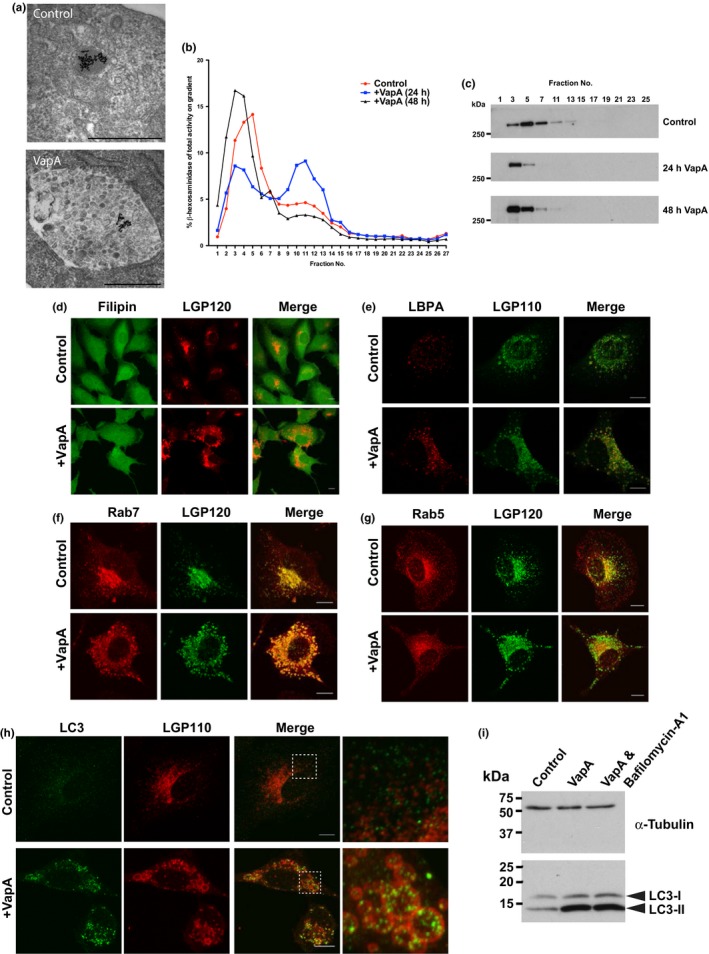
VapA feeding to cells induces the formation of large endolysosomes. (a) Cells were pulse‐chased with 10 nm BSA‐gold before feeding cells with or without 100 μg/ml VapA protein for 24 hr. Cells were fixed and processed for electron microscopy. Electron micrographs show the flocculated BSA‐gold in control and VapA‐treated cells. Scale bars = 500 nm. (b) Normal Rat Kidney (NRK) cells (treated with or without VapA protein for up to 48 hr) were lysed using nitrogen cavitation and the lysates subjected to fractionation on a 10%–16% Nycodenz gradient. Fractions were taken from the bottom of the gradient and analyzed for ß‐hexosaminidase activity. (c) Gradient fractions (as in **B**) were western blotted for the late‐endosomal marker ciMPR. (d–h) NRK cells were incubated with or without 100 μg/ml recombinant VapA‐His_6_ for 24 hr. Cells were then fixed and labeled with fillipin and LGP120 (d), LBPA and LGP110 (**e**), Rab7 and LGP120 (f), Rab5 and LGP120 (g), and LC3 and LGP110 (h). Antibodies were visualized by labeling with fluorescently labeled secondary antibodies. Scale bars, 10 μm. All images represent maximum intensity z‐projections. The images in (h**)** were taken using a Zeiss 880 confocal microscope and had additional Arrayscan processing. The right hand panels of (h) are magnifications of the indicated regions of the merge panels. (i) Western blotting of lysates for α‐tubulin and LC3 of cells treated with or without 100 μg/ml VapA‐His_6_ or 100 μg/ml VapA‐His_6_ and 100 nmol/L Bafilomycin‐A1 for 24 hr

To understand further the nature of the swollen lysosomal compartment, NRK cells were pulse‐chased with 10 nm BSA‐gold and the cells were fixed for conventional electron microscopy. Electron micrographs of control cells show the BSA‐gold in electron dense structures that have previously been characterized as lysosomes (Bright et al., 1997) (Figure 5a). In cells fed VapA protein, the BSA‐gold was found in large electron lucent structures (up to 1 μm across) containing numerous intralumenal vesicles (Figure 5a). The fusion of a lysosome with a late endosome forms an endolysosome (EL), and these data suggested that VapA might be interfering with the EL, trapping material in an endolysosomal‐like compartment, and thus preventing lysosome reformation. To test this hypothesis, we fractionated NRK cells on a Nycodenz gradient and showed that 24 hr after feeding cells VapA, the lysosomal acid hydrolase ß‐hexosaminidase partially shifted to a less‐dense region of the gradient (Figure 5b). At longer time points (48 hr), this shift in density was not observed, which corresponds with a diminished swollen phenotype seen at this time (see later, Figure 7). If VapA was trapping material in an EL, then we might have observed the late‐endosomal marker ciMPR comigrating with the pool of less‐dense ß‐hexosaminidase‐positive material at 24 hr. However, the converse was observed and the ciMPR was seen to migrate to a denser part of the gradient (Figure 5c). This is consistent with immunofluorescence data, where we do not see an increase in the colocalization between late endosomes and lysosomes in the presence of VapA (Figure 4e). However, we cannot rule out that the ciMPR can be retrieved from the swollen compartments. The idea that there is still membrane flow out of these swollen structures is confirmed by the observation that there was no cholesterol accumulation (as assessed by filipin staining) in these compartments (Figure 5d). Further characterization of the swollen structures showed that there was an increase in the colocalization between lysosomes (labeled with LGP110, the rat equivalent of human LAMP2) and the late‐endosomal unconventional phospholipid lysobisphosphatidic acid (LBPA) (Figure 5e, Fig. S5). The swollen compartments were also tightly associated with the late‐endosomal Rab protein Rab7, with very little Rab7 seen in the cytoplasm compared to the control cells (Figure 5f, Fig. S5). Consistent with earlier EEA1 data (Fig. S1), there was no visible effect on the early‐endosomal Rab protein Rab 5 (Figure 5g), although the extent of colocalization between Rab5 and LGP120 was found to increase (Fig. S5). The most dramatic effect was seen with the autophagosomal marker LC3. LC3 becomes lipidated as it associates with the lumenal side of the developing autophagosomal membrane and it eventually gets delivered to the lysosome upon autolysosome formation where it gets degraded. In cells fed VapA, there is an accumulation of LC3‐positive puncta (Figure 5h, Fig. S5) and these puncta are within the swollen LGP110‐positive structures. Western blotting showed that VapA fed cells had a large increase in the lipidated form of LC3 (LC3‐II; Figure 5i). To determine whether the increase in LC3‐II was due to an upregulation of autophagy or a lack of LC3‐II, clearance cells were also treated with bafilomycin‐A1, an inhibitor of the lysosomal V‐ATPase. Bafilomycin treatment of cells did not further increase the levels of LC3‐II (Figure 5i) indicating that the increase in LC3‐II was due to a lack of clearance and not an upregulation of autophagy. Together the accumulation of intralumenal vesicles, LBPA, Rab7, and LC3 in a LGP120/110‐positive compartment were consistent with there being a defect in ELs.

### 3.4 Endolysosomes show reduced Cathepsin B activity in the presence of VapA

**Figure 6:**
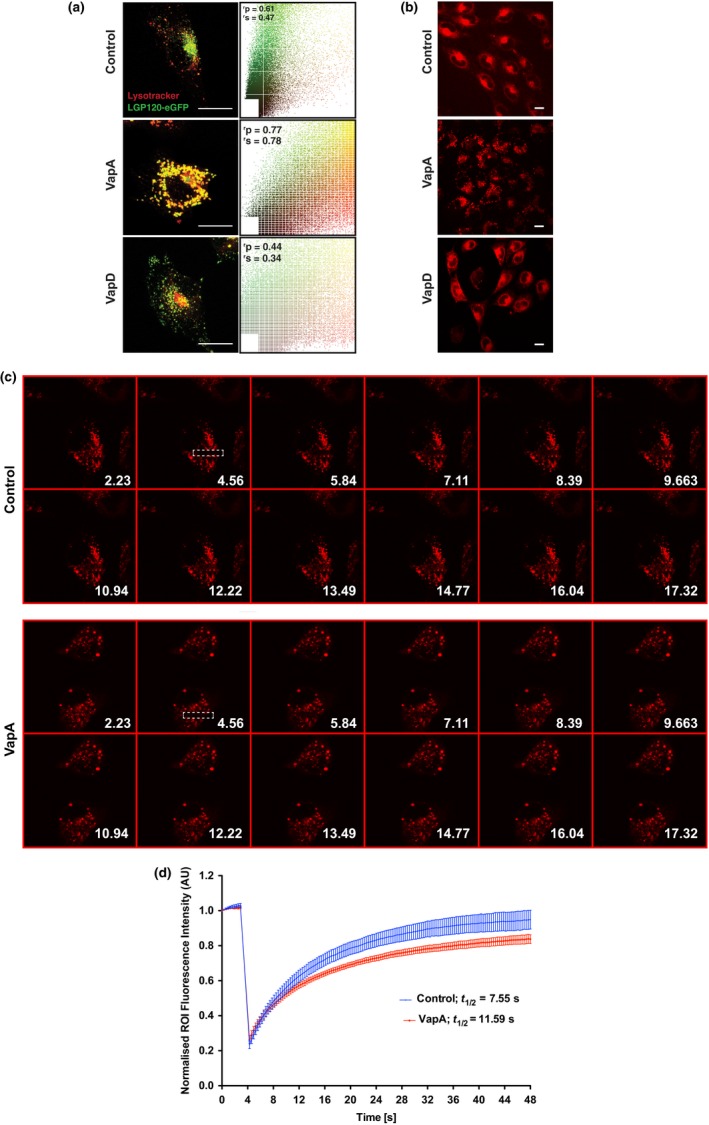
Cells fed with VapA have reduced Cathepsin B activity. (a) Normal Rat Kidney (NRK) cells stably expressing LGP120‐GFP (a kind gift from J. P. Luzio, University of Cambridge) were incubated with 100 μg/ml of recombinant VapA‐His_6_ or VapD‐His_6_ protein for 24 hr. The cells were then incubated with LysoTracker for 5 min before the LysoTracker and LGP120 were visualized on a Zeiss 880 confocal microscope. Acidic compartments are shown by red, and lysosomes by green. Scale bars are 20 μm. Quantification of colocalization is shown by Pearson–Spearman correlations and scatterplots as described (French, Mills, Swarup, Bennett, & Pridmore, 2008). (b) NRK cells were incubated with or without 100 μg/ml recombinant VapA or VapD protein for 24 hr. Cells were then incubated with Cathepsin B MagicRed substrate for 10 min before imaging the site of active Cathepsin B using an Andor Revolution XD spinning disk microscopes (c) Representative images of Magic Red Cathepsin B substrate in control cells and cells fed 100 μg/ml of recombinant VapA for 24 hr before and after photobleaching. The photobleaching ROI is shown by boxed areas. Timings are in seconds after the start of image acquisition. (d) Quantification of fluorescence recovery after photobleaching (FRAP) data as seen in c. Data are the means ± SEM from 7 to 9 cells for each condition

The LC3 result suggests that there is a defect in the hydrolase activity in the swollen EL compartments. While we have not directly measured the pH of these swollen compartments, the swollen compartments still accumulate LysoTracker, indicating that the swollen ELs are at least acidic (LysoTracker accumulates in compartments where the pH <6.5) (Figure 6a). Indeed, in control cells and cells fed VapD, there are lysosomes that are not labeled with LysoTracker, but in cells fed VapA, there are very few LGP120‐positive structures that are not labeled by LysoTracker (Figure 6a), again indicating the loss of regular lysosomes in VapA‐fed cells. Assessing if there are any subtle changes in the pH of the ELs compared to control cells is difficult to measure since in control cells there would be few ELs. We therefore assessed the hydrolase activity in cells fed with VapA using the Magic Red Cathepsin B substrate (MRCBS), which is a substrate for Cathepsin B that becomes fluorescent upon cathepsin B cleavage (Pryor, 2012). In control cells and cells fed with VapA or VapD, there was a good MRCBS signal, with the MRCBS seen in aggregated structures in VapA‐fed cells (Figure 6b). We then performed fluorescence recovery after photobleaching (FRAP) experiments in control and VapA‐fed cells (Figure 6c). In VapA‐fed cells, there is a 35% reduction in the rate of recovery of MRCBS fluorescence after photobleaching, indicating a reduction in cathepsin B activity (Figure 6d). If other acid hydrolases also show this reduced hydrolytic activity, then this could explain the accumulation of LBPA, Rab7, and LC3 and a swollen phenotype akin to a lysosomal storage disorder where material delivered to the lysosome is undigested.

### 3.5 VapA induces lysosome biogenesis

**Figure 7:**
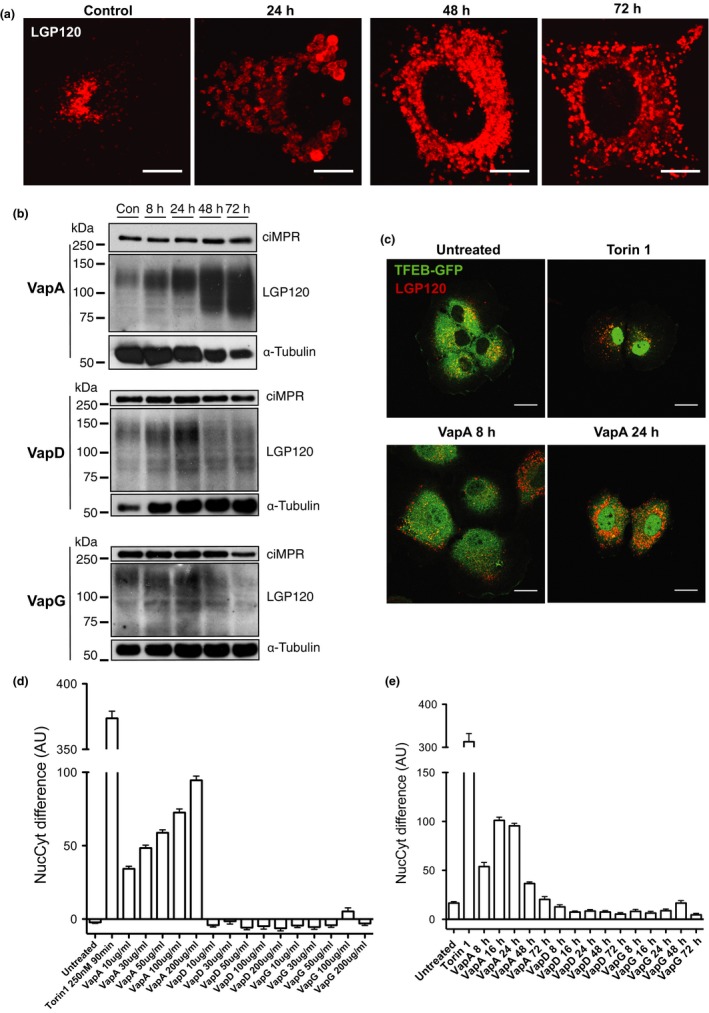
VapA induces lysosome biogenesis. (a) Normal Rat Kidney (NRK) cells were incubated with 100 μg/ml of recombinant VapA for the times indicated. Cells were then fixed and immunolabeled with LGP120 antibodies followed by fluorescently labeled secondary antibodies. Images are representative of three separate experiments. Scale bar, 10 μm. (b) NRK cells were incubated with 100 μg/ml of recombinant VapA‐His6, VapD‐His_6_, or VapG‐His_6_ for the times indicated. Cell lysates were then western blotted for lysosomes (LGP120), late endosomes (ciMPR), and the cytoskeleton (α‐tubulin). 20 μg of protein was loaded per lane. The blots are representative of three separate experiments. (c) NRK cells expressing transcription factor EB (TFEB)‐GFP were incubated with 100 μg/ml recombinant VapA for the times indicated, or with 250 nmol/L Torin 1 for 90 min (positive control). The cells were then fixed and labeled with α‐LGP120 antibodies followed by fluorescently labeled secondary antibodies. Scale bar, 10 μm. (d) NRK cells expressing TFEB‐GFP were incubated with increasing concentrations of VapA, VapD, or VapG for 24 hr. The cells were rinsed with PBS, then fixed. Cell nuclei were stained with Hoechst 33342 dye for 10 min. The cells were then scanned on a Cellomics Arrayscan plate reader to measure the translocation of TFEB‐GFP from the cytoplasm to the nucleus as described. Data are plotted as a difference of the fluorescence intensity in the nucleus compared to the cytoplasm. (e) NRK cells expressing TFEB‐GFP were incubated with 100 μg/ml of recombinant VapA, VapD, or VapG for the times indicated. The translocation of TFEB‐GFP into the nucleus was measured as in D**.** Data are means ± SEM (*n* = 1, >800 cells per condition)

When NRK cells were incubated with VapA over a longer time course, it was observed that there were more LGP120‐positive puncta between 48 hr and 72 hr (Figure 7a). Lysosome biogenesis is known to be upregulated in response to lysosomal dysfunction (Sardiello et al., 2009), which causes translocation of the transcription factor EB (TFEB) from the cytoplasm to the nucleus, resulting in a coordinated increase in expression of lysosomal genes. We hypothesized that the swollen ELs, induced by VapA, with reduced hydrolase activity would accumulate undigested material and this would result in an increase in lysosome biogenesis. NRK cells were incubated with VapA, VapD, or VapG at 100 μg/ml for 8–72 hr and the resultant lysates probed by western blotting for markers of late endosomes and lysosomes (Figure 7b). The amount LGP120, a marker of lysosomes, increased in accordance with the time cells were incubated with VapA protein. Unlike LGP120, the amount of ciMPR, a marker of late‐endosomes, did not increase. Cells incubated with VapD or VapG did not show an increase in levels of LGP120.

We utilized a quantitative cytoplasm‐to‐nucleus translocation algorithm developed by Cellomics (Williams et al., 2006) to measure the translocation of GFP‐tagged TFEB into the nucleus in response to lysosomal dysfunction. NRK cells stably expressing TFEB‐GFP were incubated with 100 μg/ml VapA for 8 hr or 24 hr. After fixing the cells, the quantitative localization of TFEB‐GFP was determined with the Cellomics ArrayScan^™^ widefield microscopy system and the morphology of lysosomes was analyzed by confocal microscopy (Figure 7c). After 8 hr of incubation with VapA, some of the TFEB‐GFP was observed to have translocated to the nucleus and swollen lysosomes could be seen. After 24 hr, most of the TFEB‐GFP had translocated to the nucleus and the severity of the lysosomal phenotype had increased, as judged by an increase in the size of lysosomal puncta. No translocation of TFEB‐GFP into the nucleus was seen in untreated control cells. In cells treated for 90 min with 250 nmol/L Torin 1 (a potent inhibitor of the mTOR complex, which is known to cause TFEB translocation into the nucleus), the majority of the TFEB‐GFP had translocated to the nucleus. TFEB‐GFP‐expressing NRK cells were incubated with increasing amounts of VapA, VapD, or VapG recombinant protein. VapA induced the translocation of TFEB‐GFP to the nucleus and this was in a dose‐dependent manner (Figure 7d). However, neither VapD nor VapG had any effect on TFEB. Translocation of TFEB‐GFP into the nucleus was also monitored over a longer time course (Figure 7e). In cells treated with VapA, there was an increase in the amount of nuclear TFEB‐GFP by 8 hr with nuclear translocation being maximal at 16–24 hr. Neither VapD nor VapG induced the translocation of TFEB into the nucleus at any time point, relative to untreated control cells. Together, these results suggest that VapA causes EL dysfunction and to compensate for the loss of lysosomes, the cell upregulates lysosome biogenesis.

## 4 Discussion

The presence of the virulence plasmid in *R. equi* is widely accepted as a prerequisite for intracellular survival and replication inside eukaryotic cells (Hondalus & Mosser, 1994). The expression of VapA in particular has been associated with virulence in vivo in both mice and foals (Giguere et al., 1999). Despite the importance of VapA in *R. equi* pathogenesis, little is known about its molecular function, although it is has been reported to reduce fusion of *R. equi* phagosomes with the lysosome (Fernandez‐Mora et al., 2005; von Bargen et al., 2009) but how is unclear. A successful intracellular pathogen is often one that can avoid, or survive being delivered to, the hydrolytic environment of the lysosome. We therefore sought to determine whether VapA disrupted the lysosome itself, as a potential mechanism for allowing *R. equi* to survive intracellularly.

Some intracellular pathogens harbor type 3 secretion systems (T3SS), which translocate effector proteins across host cell membranes. While *R. equi* possesses a type 7 secretion system (T7SS) that allows proteins to be secreted into the RCV, there is no identified T3SS in *R. equi* to secrete proteins into the host cell's cytoplasm. In this respect, *R. equi* is similar to *Mycobacterium tuberculosis*, which also lacks a known T3SS, even though effector proteins such as SapM, PtpA, and PtpB are known to act on cytoplasmic lipids and/or proteins (Margenat et al., 2015; Mascarello et al., 2013; Saleh & Belisle, 2000). Given that VapA expression in the cytoplasm of HeLa cells did not cause any morphological changes to endocytic compartments, we hypothesized that the RCV represents the site of VapA activity. To test this, we cultured cells in the presence of recombinant Vap proteins, resulting in their uptake into endocytic compartments through fluid‐phase endocytosis. We confirmed that recombinant protein accumulated in endocytic compartments (Figure 2) and that VapA is not just signaling through cell surface receptors. Importantly, we observed in a variety of cell types that when cells were fed recombinant VapA, the late‐endosomes/lysosomes rapidly became enlarged. Characterizing this phenotype further, we see large structures with intralumenal vesicles that are reminiscent of endolysosomes, with an accumulation of the late endocytic markers LBPA and Rab7 and reduced clearance of the autophagy marker LC3. These VapA‐induced swollen EL compartments are still acidic, though we cannot rule out that the pH has changed, and have reduced cathepsin B activity. The enlarged lysosome phenotype is similar to the lysosome phenotype in cells that have a lysosome storage disorder and accumulate undigested substrates. Lysosome dysfunction results in upregulation of lysosome biogenesis coordinated via the transcription factor EB (TFEB) (Sardiello et al., 2009; Settembre et al., 2012). We found that VapA caused lysosomal biogenesis as assessed by translocation of TFEB into the cell nucleus and western blotting for the lysosomal marker LGP120 in rat cells. The upregulation of lysosome biogenesis is a consequence of lysosome disruption. In our system, lysosomes would be lost as they become consumed in the formation of perturbed endolysosomes. This is consistent with the loss of lysosomes seen in *R. equi*‐infected cells (Hietala & Ardans, 1987; Zink et al., 1987). However, we then see lysosome biogenesis, which indicates a loss of lysosome function. Lysosome biogenesis is not seen in *R. equi*‐infected macrophages since the macrophages undergo necrosis first, in a VapA‐independent manner (von Bargen et al., 2009). But we do see lysosome biogenesis in J774.2 cells when fed VapA alone (data not shown).

By disrupting endolysosome function, which is essentially the phagolysosomal compartment, VapA can reduce the impact of lysosomal hydrolases and therefore can create an intracellular niche that aids *R. equi* survival. It is possible that *R. equi* phagosomes do indeed fuse with a few lysosomes, but eventually the phagolysosome becomes non‐fusogenic to further lysosomal fusion, which would show up as an overall lack of delivery to many lysosomes. Our studies have only examined the role of VapA, since it is essential for virulence, but this does not preclude other factors that may aid *R. equi* virulence. For instance, similar to *M. tuberculosis*,* R. equi* has a hydrophobic cell wall containing unusual mycolic acid‐containing glycolipids such as trehalose dimycolate and lipoarabinomannan (Alvarez, 2010). It has been shown that mannose‐capped lipoarabinomannan disrupts traffic between the trans‐Golgi network and the *M. tuberculosis* phagosome, resulting in the exclusion of the V‐ATPase (Fratti, Chua, Vergne, & Deretic, 2003). These lipids may play a similar role in altering the acidification of the RCV.

**Figure 8:**
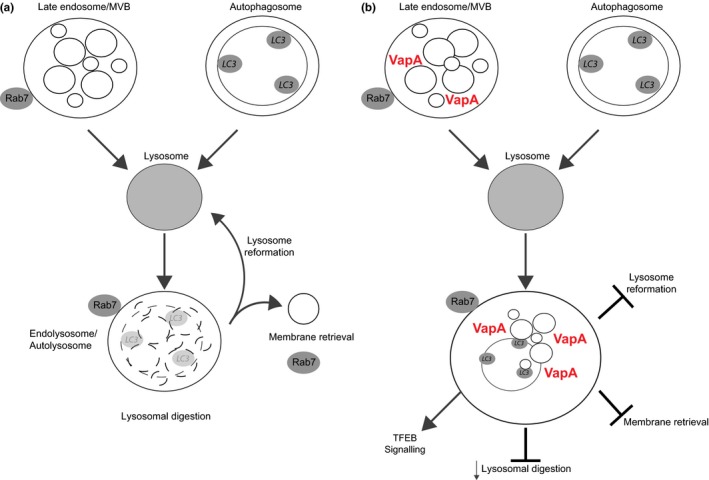
Model of VapA action on the late endocytic pathway. (a) In normal cells, late endosomes and/or autophagosomes fuse with lysosomes to form an endolysosome (EL) or an autolysosome (AL). The material delivered to the lysosome is then digested and the lysosome re‐forms from the EL/AL compartment. (b) VapA reduces the hydrolase activity in the EL/AL and material delivered to the lysosome accumulates. Lysosomes cannot be re‐formed from the perturbed EL/AL causing a loss of lysosomes. The lack of functional lysosomes is sensed by the cell and transcription factor EB (TFEB) signaling promotes lysosome biogenesis

Our data show that VapA perturbs endolysosomes and we therefore suggest that the physiologically altered ELs are unable to digest lumenal material and subsequently the compartment does not condense to re‐form lysosomes. The net result is an accumulation of undigested material and a loss of terminal lysosomes as they become consumed into the ELs. The loss of lysosomes and lysosomal function is sensed by the cell resulting in TFEB translocation to the nucleus and an increase in lysosome biogenesis (Figure 8).

Due to the close homology in the Vaps in both sequence and structure, we considered the possibility that other Vap proteins may be able to induce the same effects as VapA. Interestingly, when NRK cells were incubated with either VapD or VapG, no changes to lysosome morphology were seen and lysosomal staining was indistinguishable from that of control cells. Although it is possible that the other Vap proteins act synergistically or are somehow modified or regulated by the activity of VapA, we found that when cells were incubated with VapD and VapG in combination, they had no effect on lysosomes (data not shown). It therefore remains unclear what the function of these additional Vaps is, considering that expression of these proteins is induced during intracellular growth (Byrne et al., 2001; Ren & Prescott, 2003) and under the low pH conditions encountered in the macrophage phagosome, (Benoit, Benachour, Taouji, Auffray, & Hartke, 2001).

We also investigated which regions of VapA were responsible for its effect on lysosomes. Surprisingly, it was found that the C‐terminal core of VapA alone was sufficient to induce the swollen EL structures. This was confirmed by the fact that a VapA N‐terminus/VapD core chimeric protein had no effect on lysosome morphology. Our data strongly suggest that the activity of VapA can be attributed to the core barrel structure alone. The structure of the Vap proteins has no obvious ligand‐binding sites or grooves and gives no hints as to its function. As only late endocytic compartments appear to be affected, it is tempting to speculate that VapA requires a decrease in pH or needs to be proteolytically processed to become active. Alternatively, VapA may interact with a host‐cell component only found within the lumen of late endocytic compartments.

Together our data provide the first evidence for a likely function for VapA, namely disruption of endolysosomes, and this is likely to be a key mechanism for intracellular survival of *R. equi*. Despite the high degree of sequence homology between the Vap proteins, only VapA can disrupt the function of lysosomes. Further analysis of the differences in the C‐terminal core of VapA compared to other Vap proteins should be a fruitful future route toward discovering the precise mechanism of action of this virulence protein.

## Conflict of Interest

There are no conflicts of interest.

## References

[mbo3416-bib-0001] Alvarez, H. C. M . (2010). Biology of Rhodococcus. Heidelberg: Springer.

[mbo3416-bib-0002] Benoit, S. , Benachour, A. , Taouji, S. , Auffray, Y. , & Hartke, A. (2001). Induction of vap genes encoded by the virulence plasmid of Rhodococcus equi during acid tolerance response. Research in Microbiology, 152, 439–449. 11446512 10.1016/s0923-2508(01)01217-7

[mbo3416-bib-0003] Bright, N. A. , Reaves, B. J. , Mullock, B. M. , & Luzio, J. P. (1997). Dense core lysosomes can fuse with late endosomes and are re‐formed from the resultant hybrid organelles. Journal of Cell Science, 110(Pt 17), 2027–2040. 9378754 10.1242/jcs.110.17.2027

[mbo3416-bib-0004] Burton, A. J. , Giguère, S. , Sturgill, T. L. , Berghaus, L. J. , Slovis, N. M. , Whitman, J. L. , … Cohen, N. D . (2013). Macrolide‐ and rifampin‐resistant Rhodococcus equi on a horse breeding farm, Kentucky, USA. Emerging Infectious Diseases, 19, 282–285. 23347878 10.3201/eid1902.121210 PMC3559061

[mbo3416-bib-0005] Byrne, B. A. , Prescott, J. F. , Palmer, G. H. , Takai, S. , Nicholson, V. M. , Alperin, D. C. , & Hines, S. A. (2001). Virulence plasmid of Rhodococcus equi contains inducible gene family encoding secreted proteins. Infection and Immunity, 69, 650–656. 11159951 10.1128/IAI.69.2.650-656.2001 PMC97935

[mbo3416-bib-0006] Cauchard, S. , Giguère, S. , Venner, M. , Muscatello, G. , Cauchard, J. , Cohen, N. D. , … Vázquez‐Boland, J. (2013). Rhodococcus equi research 2008‐2012: Report of the Fifth International Havemeyer Workshop. Equine Veterinary Journal, 45, 523–526. 23909447 10.1111/evj.12103

[mbo3416-bib-0007] Coulson, G. B. , Miranda‐CasoLuengo, A. A. , Miranda‐CasoLuengo, R. , Wang, X. , Oliver, J. , Willingham‐Lane, J. M. , … Hondalus, M. K. (2015). Transcriptome reprogramming by plasmid‐encoded transcriptional regulators is required for host niche adaption of a macrophage pathogen. Infection and Immunity, 83, 3137–3145. 26015480 10.1128/IAI.00230-15 PMC4496601

[mbo3416-bib-0008] Fernandez‐Mora, E. , Polidori, M. , Luhrmann, A. , Schaible, U. E. , & Haas, A. (2005). Maturation of Rhodococcus equi‐containing vacuoles is arrested after completion of the early endosome stage. Traffic, 6, 635–653. 15998320 10.1111/j.1600-0854.2005.00304.x

[mbo3416-bib-0009] Fratti, R. A. , Chua, J. , Vergne, I. , & Deretic, V. (2003). Mycobacterium tuberculosis glycosylated phosphatidylinositol causes phagosome maturation arrest. Proceedings of the National Academy of Sciences U S A, 100, 5437–5442. 10.1073/pnas.0737613100 PMC154363 12702770

[mbo3416-bib-0010] French, A. P. , Mills, S. , Swarup, R. , Bennett, M. J. , & Pridmore, T. P. (2008). Colocalization of fluorescent markers in confocal microscope images of plant cells. Nature Protocols, 3, 619–628. 18388944 10.1038/nprot.2008.31

[mbo3416-bib-0011] Geerds, C. , Wohlmann, J. , Haas, A. , & Niemann, H. H. (2014). Structure of Rhodococcus equi virulence‐associated protein B (VapB) reveals an eight‐stranded antiparallel beta‐barrel consisting of two Greek‐key motifs. Acta Crystallographica Section F: Structural Biology Communications, 70, 866–871. 25005079 10.1107/S2053230X14009911 PMC4089522

[mbo3416-bib-0012] Giguère, S. , Cohen, N. D. , Chaffin, M. K. , Slovis, N. M. , Hondalus, M. K. , Hines, S. A. , & Prescott, J. F. (2011). Diagnosis, treatment, control, and prevention of infections caused by Rhodococcus equi in foals. Journal of Veterinary Internal Medicine, 25, 1209–1220. 22092608 10.1111/j.1939-1676.2011.00835.x

[mbo3416-bib-0013] Giguere, S. , Hondalus, M. K. , Yager, J. A. , Darrah, P. , Mosser, D. M. , & Prescott, J. F. (1999). Role of the 85‐kilobase plasmid and plasmid‐encoded virulence‐associated protein A in intracellular survival and virulence of Rhodococcus equi. Infection and Immunity, 67, 3548–3557. 10377138 10.1128/iai.67.7.3548-3557.1999 PMC116543

[mbo3416-bib-0014] Gonzalez‐Iglesias, P. , Scortti, M. , MacArthur, I. , Hapeshi, A. , Rodriguez, H. , Prescott, J. F. , & Vazquez‐Boland, J. A. (2014). Mouse lung infection model to assess Rhodococcus equi virulence and vaccine protection. Veterinary Microbiology, 172, 256–264. 24852140 10.1016/j.vetmic.2014.03.026

[mbo3416-bib-0101] Gordon, D. E. , Mirza, M. , Sahlender, D. A. , Jakovleska, J. , & Peden, A. A . (2009). Coiled‐coil interactions are required for post‐Golgi R‐SNARE trafficking. EMBO Reports, 10, 851–856. 19557002 10.1038/embor.2009.96 PMC2726663

[mbo3416-bib-0015] Hayes, D. Jr , Diaz‐Guzman, E. , & Hoopes, C. W. (2011). Rhodococcus equi infection after lung transplantation. Respiratory Care, 56, 1605–1607. 21513607 10.4187/respcare.01132

[mbo3416-bib-0016] Hietala, S. K. , & Ardans, A. A. (1987). Interaction of Rhodococcus equi with phagocytic cells from R. equi‐exposed and non‐exposed foals. Veterinary Microbiology, 14, 307–320. 3672873 10.1016/0378-1135(87)90118-0

[mbo3416-bib-0017] Hondalus, M. K. , & Mosser, D. M. (1994). Survival and replication of Rhodococcus equi in macrophages. Infection and Immunity, 62, 4167–4175. 7927672 10.1128/iai.62.10.4167-4175.1994 PMC303092

[mbo3416-bib-0018] Jain, S. , Bloom, B. R. , & Hondalus, M. K. (2003). Deletion of vapA encoding Virulence Associated Protein A attenuates the intracellular actinomycete Rhodococcus equi. Molecular Microbiology, 50, 115–128. 14507368 10.1046/j.1365-2958.2003.03689.x

[mbo3416-bib-0019] Kakuda, T. , Hirota, T. , Takeuchi, T. , Hagiuda, H. , Miyazaki, S. , & Takai, S. (2014). VirS, an OmpR/PhoB subfamily response regulator, is required for activation of vapA gene expression in Rhodococcus equi. BMC Microbiology, 14, 243. 25281192 10.1186/s12866-014-0243-1 PMC4190465

[mbo3416-bib-0020] Khan, M. Y. , Ali, S. , & Baqi, S. (2013). Rhodococcus equi pneumonia in a live related renal transplant recipient. Journal of Pakistan Medical Association, 63, 635–638. 23757997

[mbo3416-bib-0021] Letek, M. , Gonzalez, P. , Macarthur, I. , Rodriguez, H. , Freeman, T. C. , Valero‐Rello, A. , … Vázquez‐Boland, J. A. (2010). The genome of a pathogenic rhodococcus: Cooptive virulence underpinned by key gene acquisitions. PLoS Genetics, 6, e1001145. 20941392 10.1371/journal.pgen.1001145 PMC2947987

[mbo3416-bib-0022] Luhrmann, A. , Mauder, N. , Sydor, T. , Fernandez‐Mora, E. , Schulze‐Luehrmann, J. , Takai, S. , & Haas, A. (2004). Necrotic death of Rhodococcus equi‐infected macrophages is regulated by virulence‐associated plasmids. Infection and Immunity, 72, 853–862. 14742529 10.1128/IAI.72.2.853-862.2004 PMC321572

[mbo3416-bib-0023] Margenat, M. , Labandera, A. M. , Gil, M. , Carrion, F. , Purificacao, M. , Razzera, G. , … Villarino, A . (2015). New potential eukaryotic substrates of the mycobacterial protein tyrosine phosphatase PtpA: Hints of a bacterial modulation of macrophage bioenergetics state. Scientific Reports, 5, 8819. 25743628 10.1038/srep08819 PMC5390082

[mbo3416-bib-0024] Mascarello, A. , Mori, M. , Chiaradia‐Delatorre, L. D. , Menegatti, A. C. , Delle Monache, F. , Ferrari, F. , … Botta, M. (2013). Discovery of Mycobacterium tuberculosis protein tyrosine phosphatase B (PtpB) inhibitors from natural products. PLoS ONE, 8, e77081. 24155919 10.1371/journal.pone.0077081 PMC3796549

[mbo3416-bib-0025] Muscatello, G. , Anderson, G. A. , Gilkerson, J. R. , & Browning, G. F. (2006). Associations between the ecology of virulent Rhodococcus equi and the epidemiology of R. equi pneumonia on Australian thoroughbred farms. Applied and Environment Microbiology, 72, 6152–6160. 10.1128/AEM.00495-06 PMC1563629 16957241

[mbo3416-bib-0026] Nath, S. R. , Mathew, A. P. , Mohan, A. , & Anila, K. R. (2013). Rhodococcus equi granulomatous mastitis in an immunocompetent patient. Journal of Medical Microbiology, 62, 1253–1255. 23699061 10.1099/jmm.0.054346-0

[mbo3416-bib-0027] Okoko, T. , Blagova, E. V. , Whittingham, J. L. , Dover, L. G. , & Wilkinson, A. J. (2015). Structural characterisation of the virulence‐associated protein VapG from the horse pathogen Rhodococcus equi. Veterinary Microbiology, 179, 42–52. 25746683 10.1016/j.vetmic.2015.01.027 PMC4518536

[mbo3416-bib-0028] Pryor, P. R. (2012). Analyzing lysosomes in live cells. Methods in Enzymology, 505, 145–157. 22289452 10.1016/B978-0-12-388448-0.00016-4

[mbo3416-bib-0029] Pryor, P. R . (2015). Isolation of Lysosomes from Rat Tissue and Tissue Culture Cells. Cold Spring Harbor, New York: Cold Spring Harbor Laboratory Press.

[mbo3416-bib-0030] Ren, J. , & Prescott, J. F. (2003). Analysis of virulence plasmid gene expression of intra‐macrophage and in vitro grown Rhodococcus equi ATCC 33701. Veterinary Microbiology, 94, 167–182. 12781484 10.1016/s0378-1135(03)00099-3

[mbo3416-bib-0031] Ren, J. , & Prescott, J. F. (2004). The effect of mutation on Rhodococcus equi virulence plasmid gene expression and mouse virulence. Veterinary Microbiology, 103, 219–230. 15504593 10.1016/j.vetmic.2004.08.005

[mbo3416-bib-0032] Robert, X. , & Gouet, P. (2014). Deciphering key features in protein structures with the new ENDscript server. Nucleic Acids Research, 42, W320–W324. 24753421 10.1093/nar/gku316 PMC4086106

[mbo3416-bib-0033] Russell, D. A. , Byrne, G. A. , O'Connell, E. P. , Boland, C. A. , & Meijer, W. G. (2004). The LysR‐type transcriptional regulator VirR is required for expression of the virulence gene vapA of Rhodococcus equi ATCC 33701. Journal of Bacteriology, 186, 5576–5584. 15317761 10.1128/JB.186.17.5576-5584.2004 PMC516814

[mbo3416-bib-0034] Saleh, M. T. , & Belisle, J. T. (2000). Secretion of an acid phosphatase (SapM) by Mycobacterium tuberculosis that is similar to eukaryotic acid phosphatases. Journal of Bacteriology, 182, 6850–6853. 11073936 10.1128/jb.182.23.6850-6853.2000 PMC111434

[mbo3416-bib-0035] Sardiello, M. , Palmieri, M. , di Ronza, A. , Medina, D. L. , Valenza, M. , Gennarino, V. A. , … Ballabio, A. (2009). A gene network regulating lysosomal biogenesis and function. Science, 325, 473–477. 19556463 10.1126/science.1174447

[mbo3416-bib-0036] Settembre, C. , Zoncu, R. , Medina, D. L. , Vetrini, F. , Erdin, S. , Erdin, S. , et al. (2012). A lysosome‐to‐nucleus signalling mechanism senses and regulates the lysosome via mTOR and TFEB. EMBO Journal, 31, 1095–1108. 22343943 10.1038/emboj.2012.32 PMC3298007

[mbo3416-bib-0037] Takai, S. , Sekizaki, T. , Ozawa, T. , Sugawara, T. , Watanabe, Y. , & Tsubaki, S. (1991). Association between a large plasmid and 15‐ to 17‐kilodalton antigens in virulent Rhodococcus equi. Infection and Immunity, 59, 4056–4060. 1937765 10.1128/iai.59.11.4056-4060.1991 PMC258996

[mbo3416-bib-0038] Topino, S. , Galati, V. , Grilli, E. , & Petrosillo, N. (2010). Rhodococcus equi infection in HIV‐infected individuals: Case reports and review of the literature. AIDS patient care and STDs, 24, 211–222. 20377432 10.1089/apc.2009.0248

[mbo3416-bib-0039] Toyooka, K. , Takai, S. , & Kirikae, T. (2005). Rhodococcus equi can survive a phagolysosomal environment in macrophages by suppressing acidification of the phagolysosome. Journal of Medical Microbiology, 54, 1007–1015. 16192430 10.1099/jmm.0.46086-0

[mbo3416-bib-0040] Vazquez‐Boland, J. A. , Giguere, S. , Hapeshi, A. , MacArthur, I. , Anastasi, E. , & Valero‐Rello, A. (2013). Rhodococcus equi: The many facets of a pathogenic actinomycete. Veterinary Microbiology, 167, 9–33. 23993705 10.1016/j.vetmic.2013.06.016

[mbo3416-bib-0041] von Bargen, K. , Polidori, M. , Becken, U. , Huth, G. , Prescott, J. F. , & Haas, A. (2009). Rhodococcus equi virulence‐associated protein A is required for diversion of phagosome biogenesis but not for cytotoxicity. Infection and Immunity, 77, 5676–5681. 19797071 10.1128/IAI.00856-09 PMC2786453

[mbo3416-bib-0042] Whittingham, J. L. , Blagova, E. V. , Finn, C. E. , Luo, H. , Miranda‐CasoLuengo, R. , Turkenburg, J. P. , … Wilkinson, A. J. (2014). Structure of the virulence‐associated protein VapD from the intracellular pathogen Rhodococcus equi. Acta Crystallographica. Section D, Biological Crystallography, 70, 2139–2151. 25084333 10.1107/S1399004714012632 PMC4118825

[mbo3416-bib-0043] Williams, R. G. , Kandasamy, R. , Nickischer, D. , Trask, O. J. Jr , Laethem, C. , Johnston, P. A. , & Johnston, P. A. (2006). Generation and characterization of a stable MK2‐EGFP cell line and subsequent development of a high‐content imaging assay on the Cellomics ArrayScan platform to screen for p38 mitogen‐activated protein kinase inhibitors. Methods in Enzymology, 414, 364–389. 17110203 10.1016/S0076-6879(06)14021-5

[mbo3416-bib-0044] Zink, M. C. , Yager, J. A. , Prescott, J. F. , & Fernando, M. A. (1987). Electron microscopic investigation of intracellular events after ingestion of Rhodococcus equi by foal alveolar macrophages. Veterinary Microbiology, 14, 295–305. 3672872 10.1016/0378-1135(87)90117-9

