# Supplementary figures and images for: The Rhodococcus equi virulence protein VapA disrupts endolysosome function and stimulates lysosome biogenesis
Source: Microbiologyopen. 2016 Oct 19;6(2):e00416. doi: 10.1002/mbo3.416 (PMC5387311; doi:10.1002/mbo3.416)

**A**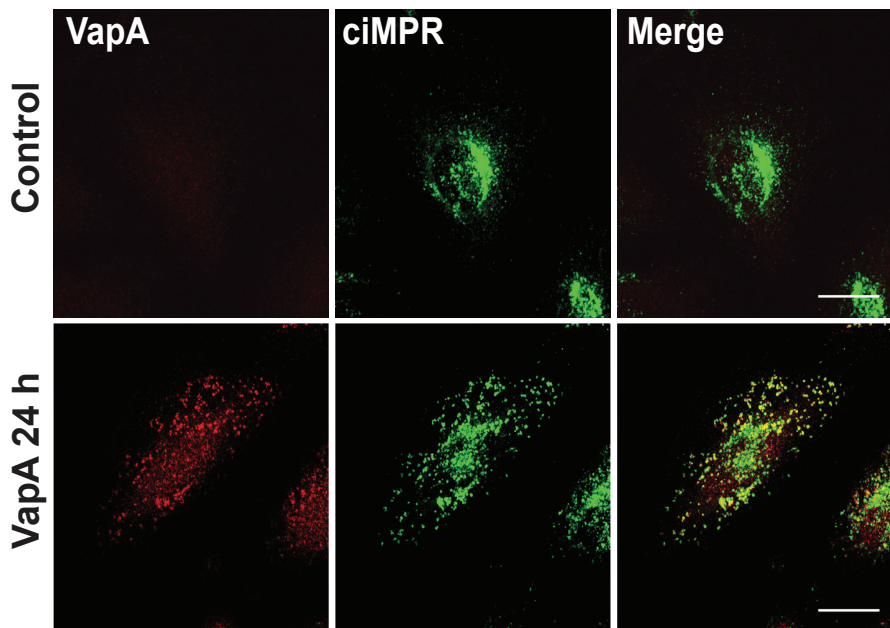**B**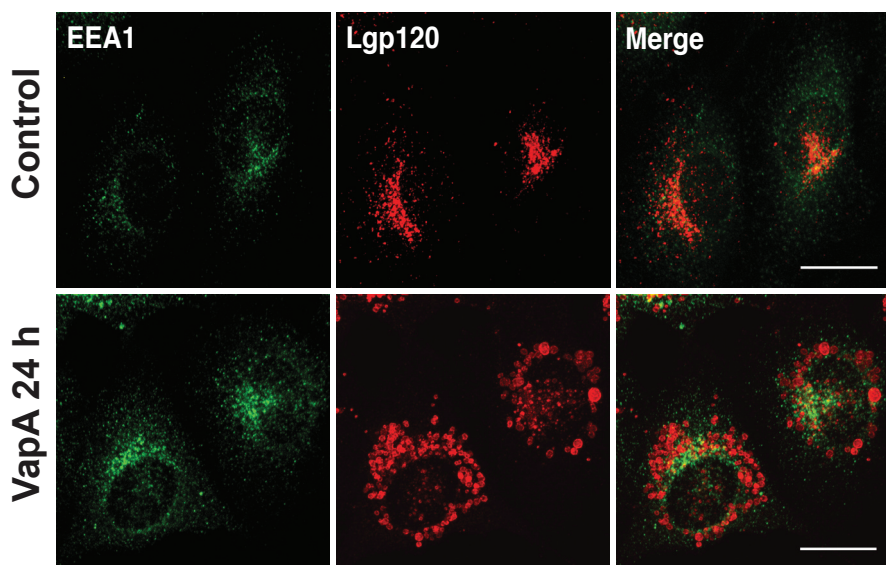**Fig. S1**

**A**

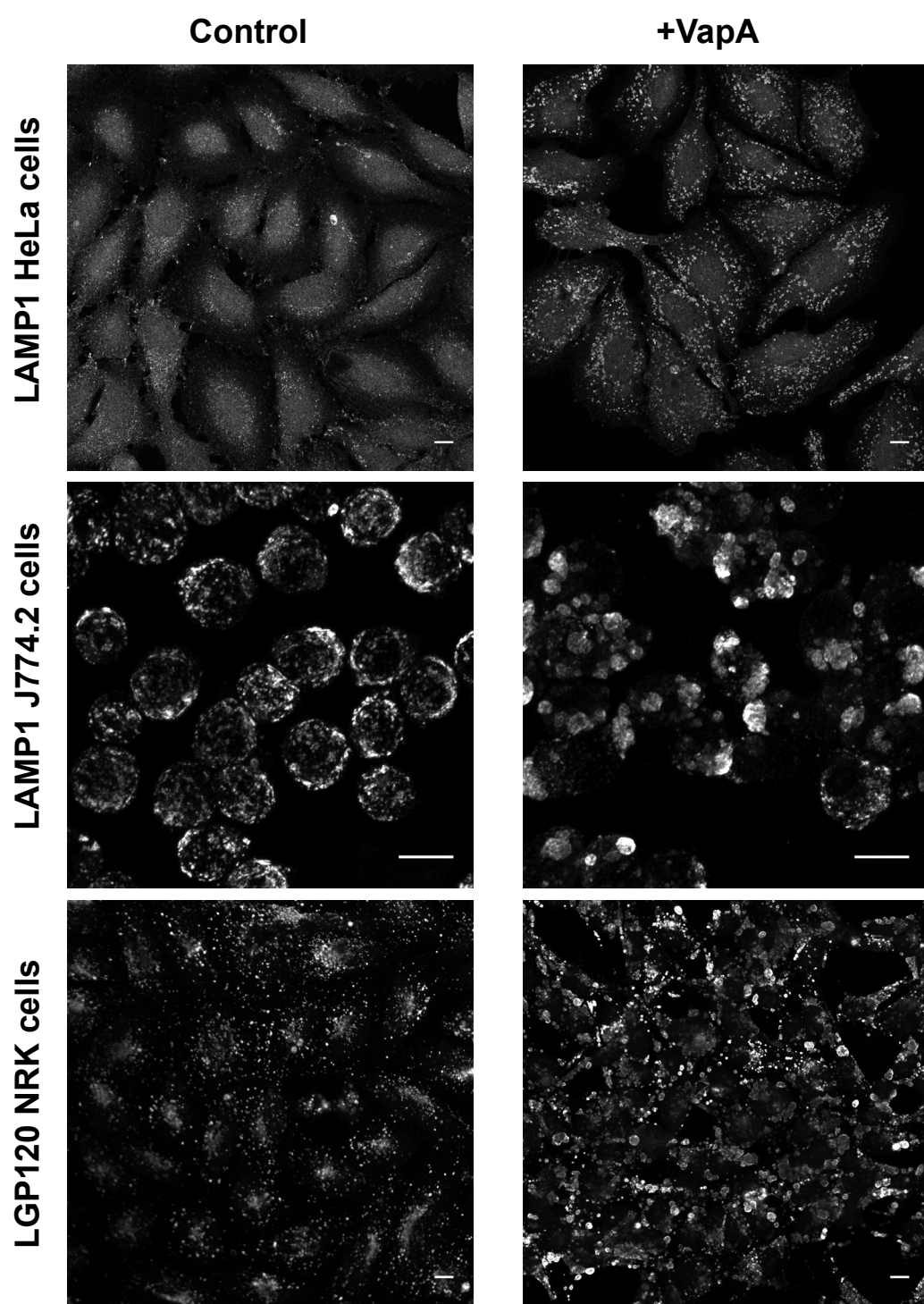

**B**

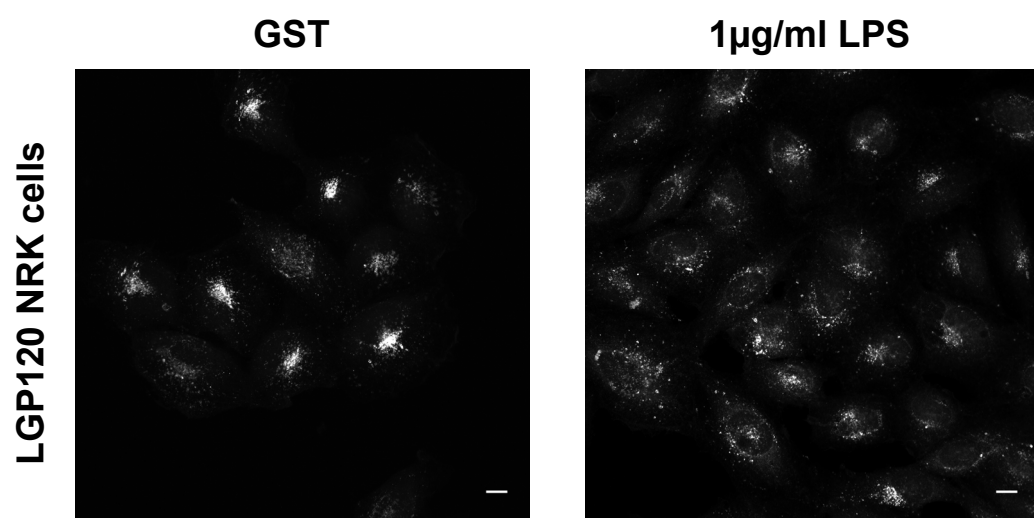

**Fig.S2**

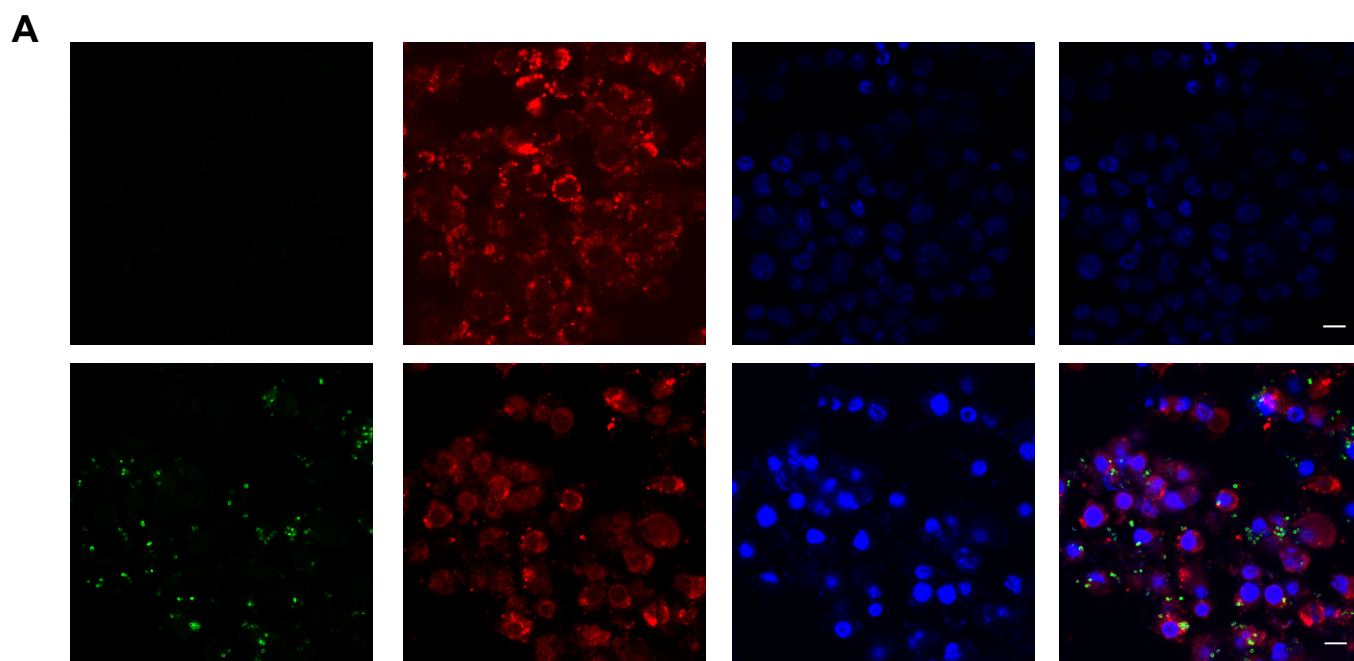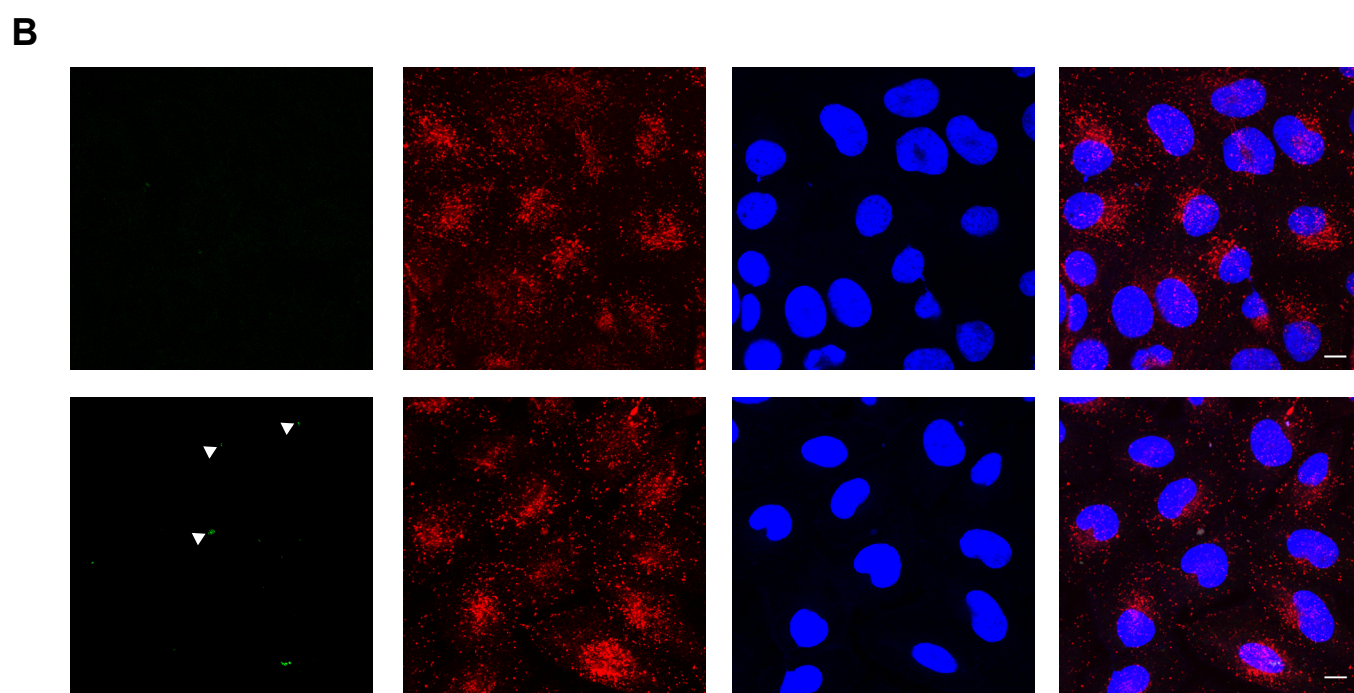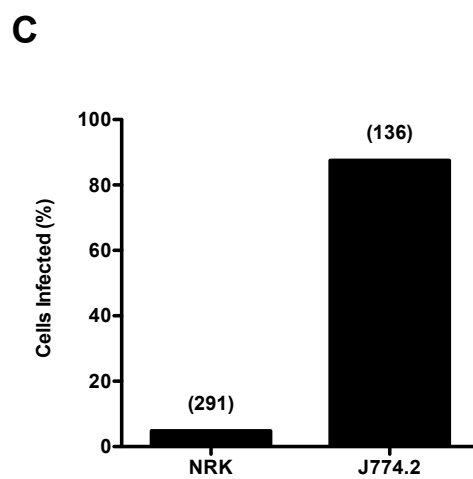

**Fig. S3**

**A****VapA**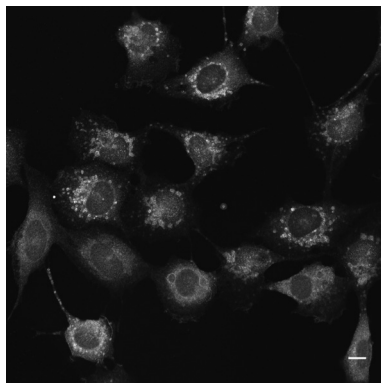**VapD**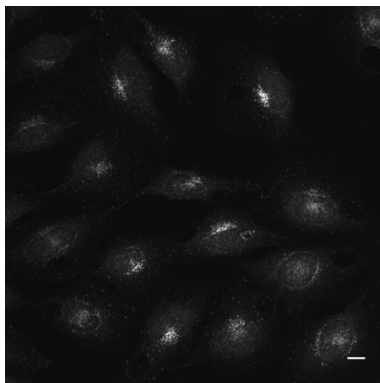**VapG**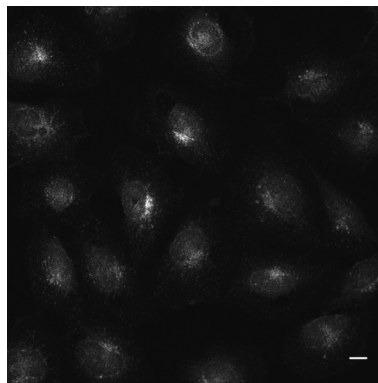**B****Control**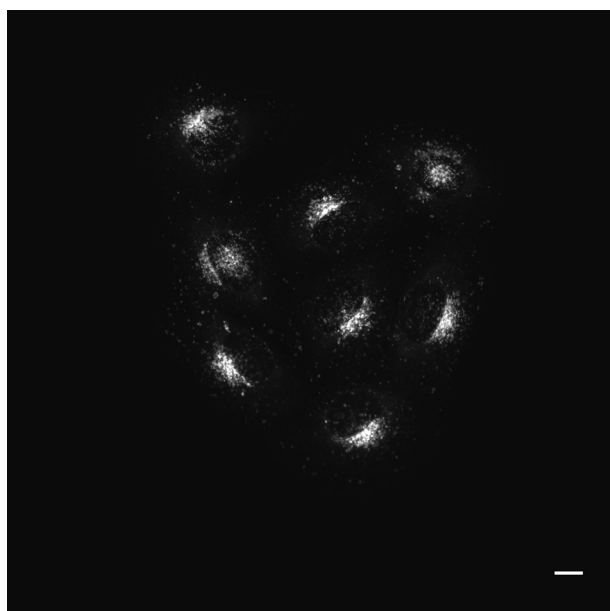**VapA Core**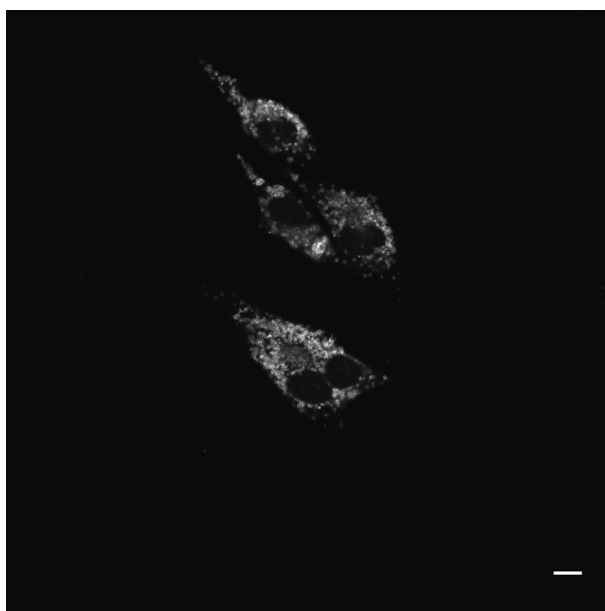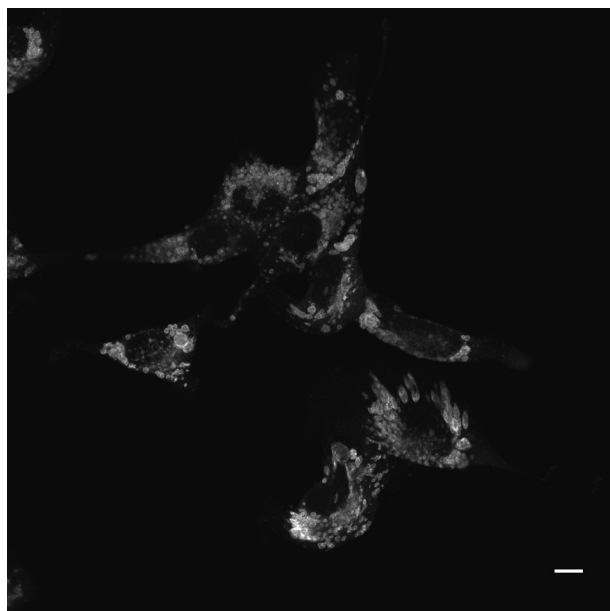**VapDA**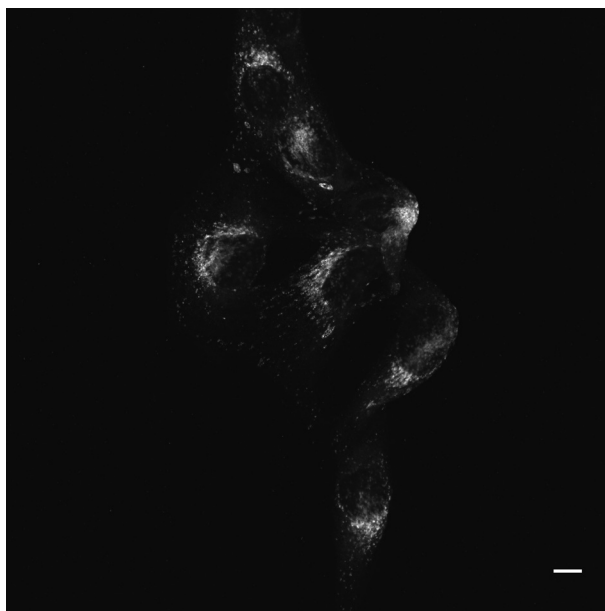**VapAD**

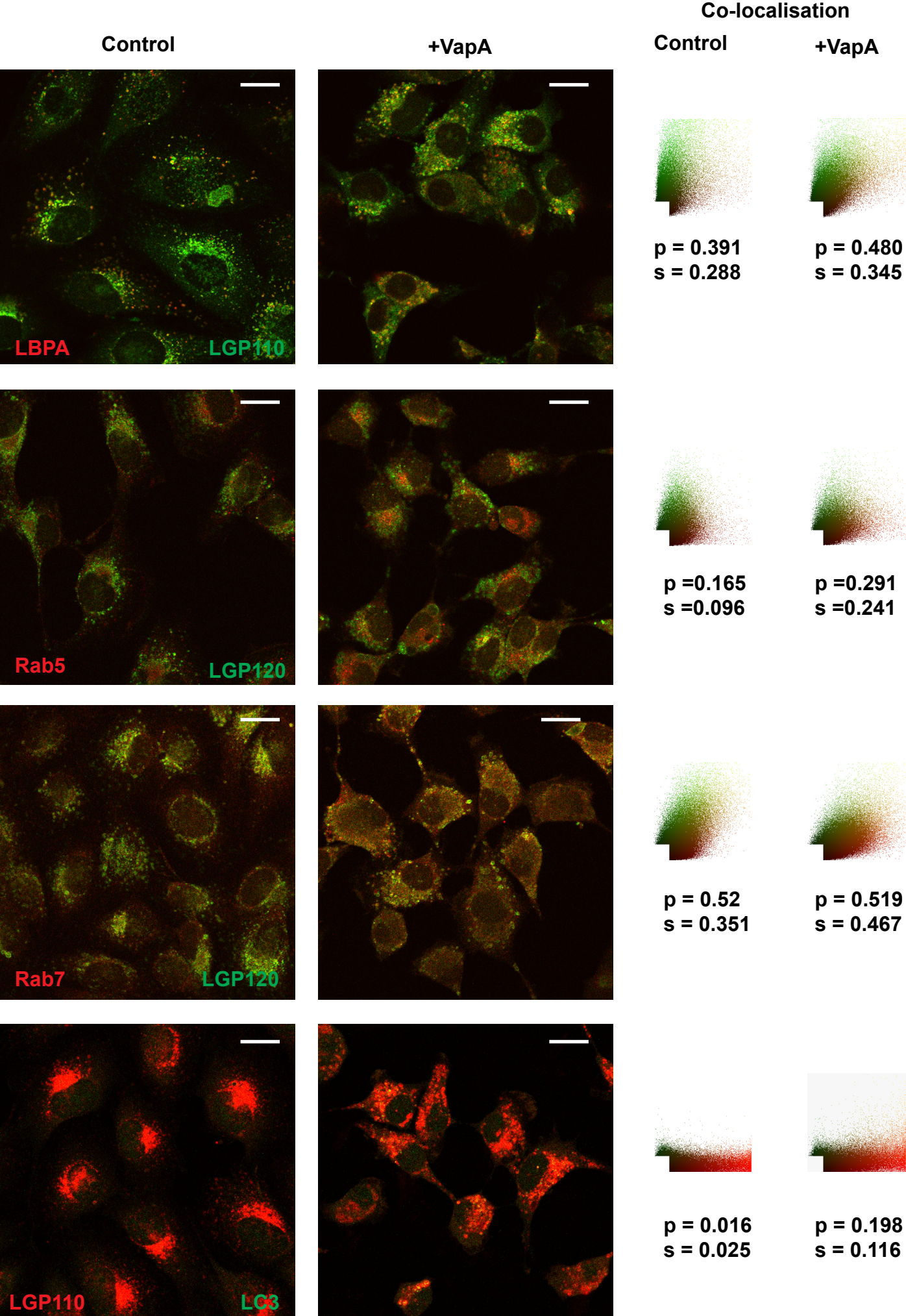

**Fig. S5**

Supplement: Supplementary file 1 [file MBO3-6-na-s001.pdf]
